# The role of the cytoskeletal proteins MreB and FtsZ in multicellular cyanobacteria

**DOI:** 10.1002/2211-5463.13016

**Published:** 2020-11-13

**Authors:** Benjamin L. Springstein, Julia Weissenbach, Robin Koch, Fenna Stücker, Karina Stucken

**Affiliations:** ^1^ Institute of General Microbiology Christian‐Albrechts University of Kiel Germany; ^2^ Present address: Faculty of Biology Technion‐Israel Institute of Technology Haifa Israel

**Keywords:** Cyanobacteria, cytoskeleton, FtsZ, morphogenesis, MreB, Stigonematales

## Abstract

Multiseriate and true‐branching cyanobacteria are at the peak of prokaryotic morphological complexity. However, little is known about the mechanisms governing multiplanar cell division and morphogenesis. Here, we study the function of the prokaryotic cytoskeletal proteins, MreB and FtsZ in *Fischerella muscicola* PCC 7414 and *Chlorogloeopsis fritschii* PCC 6912. Vancomycin and HADA labeling revealed a mixed apical, septal, and lateral trichome growth mode in *F. muscicola*, whereas *C. fritschii* exhibits septal growth. In all morphotypes from both species, MreB forms either linear filaments or filamentous strings and can interact with FtsZ. Furthermore, multiplanar cell division in *F. muscicola* likely depends on FtsZ dosage. Our results lay the groundwork for future studies on cytoskeletal proteins in morphologically complex cyanobacteria.

## Graphical abstract

Among the myriad of cyanobacterial shapes and sizes, Stigonematales show the highest complexity as they present different cell shapes and sizes within a single cyanobacterial strain. By studying the main cytoskeletal components in these cyanobacteria, we aim to understand how cells undergo these changes. We provide the first insights on the role of the cytoskeletal proteins MreB and FtsZ in cyanobacterial morphogenesis.

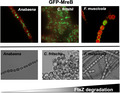

**Abbreviations**
Amp: ampicillin
BACTH: bacterial adenylate cyclase two‐hybrid
BSA: bovine serum albumin
CLB: cell lysis buffer
Cm: chloramphenicol
EDTA: ethylenediaminetetraacetic acid
EGTA: ethylene glycol‐bis(2‐aminoethylether)‐*N*,*N*,*N*′,*N*′‐tetraacetic acid
GFP: green fluorescent protein
HADA: 3‐[[(7‐Hydroxy‐2‐oxo‐2H‐1‐benzopyran‐3‐yl)carbonyl]amino]‐d‐alanine hydrochloride
HEPES: 4‐(2‐Hydroxyethyl)‐1‐piperazineethanesulfonic acid
Km: kanamycin
MAFFT: Multiple Alignment using Fast Fourier Transform
Nm: neomycin
PBS‐N: phosphate‐buffered saline with NP‐40
PBS‐T: phosphate‐buffered saline with tween
PCC: Pasteur Culture Collection
PCR: polymerase chain reaction
PG: peptidoglycan
PIC: protease inhibitor cocktail
RFP: red fluorescent protein
Van‐FL: fluorescent vancomycin

Bacterial organisms are highly diverse in cell size and shape. The bacterial cell shape is controlled by the cell wall formation, whose biosynthesis pathway is conserved among eubacteria, independently of cellular morphology [1]. This diversity in bacterial cell shape is the result of differences in the spatiotemporal organization of peptidoglycan (PG) deposition during cell wall biosynthesis [2]. A coordinated activity of peptidoglycan synthases and cytoskeletal proteins involved in PG localization determines the morphological characteristics of bacteria [3]. The localization and movement of MreB polymers correlate with PG deposition, and consequently, MreB has been described as a morphogen and determinant of cell shape in many species [4, 5, 6, 7]. For example, depletion of *mreB* expression in rod‐shaped bacteria leads to the formation of spherical cells [3, 8]. Furthermore, many coccoid bacteria and some rod‐shaped bacteria from the Rhizobiales and Actinobacteriales lack *mreB* [8, 9, 10, 11]. The growth pattern of rod‐shaped species lacking *mreB* is characterized by polar extension instead of homogeneous enlargement of the cell axis [10], as observed in *Escherichia coli* or *Bacillus subtilis* [12]. The spatial organization of MreB in rod‐shaped bacteria is either as patches perpendicular to the cell length [4, 5, 13], or as extended filaments [14] that move circumferentially along the longitudinal cell axis, driven by the PG biosynthesis machinery [5]. Additional proteins have been described to function as morphogens, also in the presence of MreB. For example, *Streptomyces'* hyphal growth depends on the polarisome and DivIVA function [15], while the crescent shape of *Caulobacter crescentus* cells is determined by the intermediate filament‐like protein Crescentin [16].

Bacterial morphogenesis is considered to be tightly related to cell division; a process governed by the prokaryotic tubulin homolog FtsZ. The function of FtsZ is to drive PG biosynthesis together with the divisome, a multiprotein complex [17]. Cell elongation and cell division are well coordinated in rod‐shaped bacteria where imbalance between the two processes may lead to altered cell shape. The FtsZ ring formation during cell division is a tightly controlled process. Changes in *ftsZ* expression level or protein localization have downstream consequences to cytokinesis as well as altered morphology and size, or even cell viability [18]. The consequences of *ftsZ* expression level have been extensively studied in *E. coli*. These studies showed that *ftsZ* depletion leads to elongated cells, while *ftsZ* overexpression leads to multiple cell divisions and the formation of minicells [19]. Further increase in *ftsZ* expression level leads to inhibition of cell division and the formation of filaments [19]. Consequently, FtsZ levels have to be tightly regulated within the cell and FtsZ levels and FtsZ protein filament dynamics were shown to be regulated by the ClpXP protease in *E. coli* [20].

Among the prokaryotes, species of the Cyanobacteria phylum encompass the largest morphological diversity, with the Stigonematales (Subsection V) representing the peak of morphological complexity [21]. Studies of MreB and FtsZ function in cyanobacteria so far revealed that these proteins may be involved in cyanobacterial phenotypic diversity. In the unicellular species *Synechococcus elongatus* PCC 7942 (*Synechococcus*), *mreB* was reported as essential where nonsegregated mutants turned coccoid. However, the mutation had a pleiotropic effect; thus, the role of MreB in *S. elongatus* remains unclear [22, 23]. In filamentous cyanobacteria, MreB appears to be involved in the maintenance of cell shape. In the heterocyst‐forming subsection IV cyanobacterium *Anabaena* sp. PCC 7120 (*Anabaena*), *mreB* knockout mutants show altered cell morphology and reveal cell wall defects [24]. Moreover, *mreB* expression level in the filamentous cyanobacterium *Fremyella diplosiphon* is responsible for the transition from rectangular to smaller and spherical cells during chromatic adaptation [25].

Studies of FtsZ showed that its gene is essential in all cyanobacteria examined so far, including *Anabaena* and the unicellular coccoid cyanobacterium *Synechocystis* sp. PCC 6803 (*Synechocystis*) [26, 27, 28, 29, 30]. Furthermore, it has been shown that the FtsZ‐driven divisome functions in the correct placement of the septal‐localized SepJ protein, which is essential for *Anabaena* multicellularity and intercellular exchange. Based upon this, it was hypothesized that the divisome has a role beyond cell division, functioning in the maintenance of *Anabaena* multicellularity and cell–cell communication [31]. In the cyanobacterium *Chroococcidiopsis* (Subsection II), which is characterized by multiplanar cell division, FtsZ was hypothesized to play a role in coordinating the timing and localization of successive cell divisions that form the baeocyte [30]. The overexpression of *ftsZ* in the rod‐shaped unicellular cyanobacterium *S. elongatus* leads to cell elongation [32], as also observed in *E. coli* [19]. In contrast, *ftsZ* overexpression in the coccoid unicellular *Synechocystis* has no impact on the cellular morphology [26], whereas depletion of other cell division genes leads to enlarged spherical cells [33, 34].

Species of the Stigonematales cyanobacteria are characterized by the presence of diverse cell shapes, cell filament (i.e. trichome) formation, and cell differentiation. Additionally, species of the Stigonematales are characterized by sophisticated restriction modification systems and thick exopolysaccharide sheaths surrounding the cells, which pose strong barriers for efficient transfer of DNA into these organisms [35]. One example of the Stigonematales is *Fischerella muscicola* PCC 7414 where vegetative cells within a mature trichome can display a mixture of cell shapes, including spherical, elliptical, and rod‐shaped cells, while cells located at the branch tips are typically tapered. The different cell forms can also vary in size, reaching a diameter of up to 8 μm [36]. Stigonematalean cyanobacteria are further characterized by an exceptional cell division program, where cells divide at a right angle to the longitudinal filament axis and in planes parallel or oblique to the primary trichomes. The multiplanar cell division in Stigonematales leads to the formation of true branches as in *F. muscicola* or to multiseriate trichomes (i.e. more than one trichome in a row) as in *Chlorogloeopsis fritschii* PCC 6912 [21, 37, 38]. Unlike *Anabaena*, *F. muscicola* and *C. fritschii* spread through the development of fast‐growing and motile linear trichomes, called hormogonia [39] that are released from mature trichomes at sites of necridia formation, which are terminally differentiated dead cells [21, 40].

Currently, no information is available on the molecular mechanisms that govern multiplanar cell division and that produce the multitude of different cell shapes in these complex cyanobacteria. It is tenable to hypothesize that multiplanar cell division in Stigonematales involves the reorganization of the cell division machinery and that the different cell shapes could be the construct of specific cell growth processes. However, data on FtsZ and MreB function and their regulation in these organisms are lacking. Here, we study the role of MreB and FtsZ in the morphological diversity of Stigonematales and Nostocales cyanobacteria. For this, we focus our effort on two representative species from the Stigonematales: *F. muscicola* that forms branching trichomes and *C. fritschii* that forms multiseriate trichomes and compare them to *Anabaena* that forms linear trichomes (Fig. 1A).

**Fig. 1:**
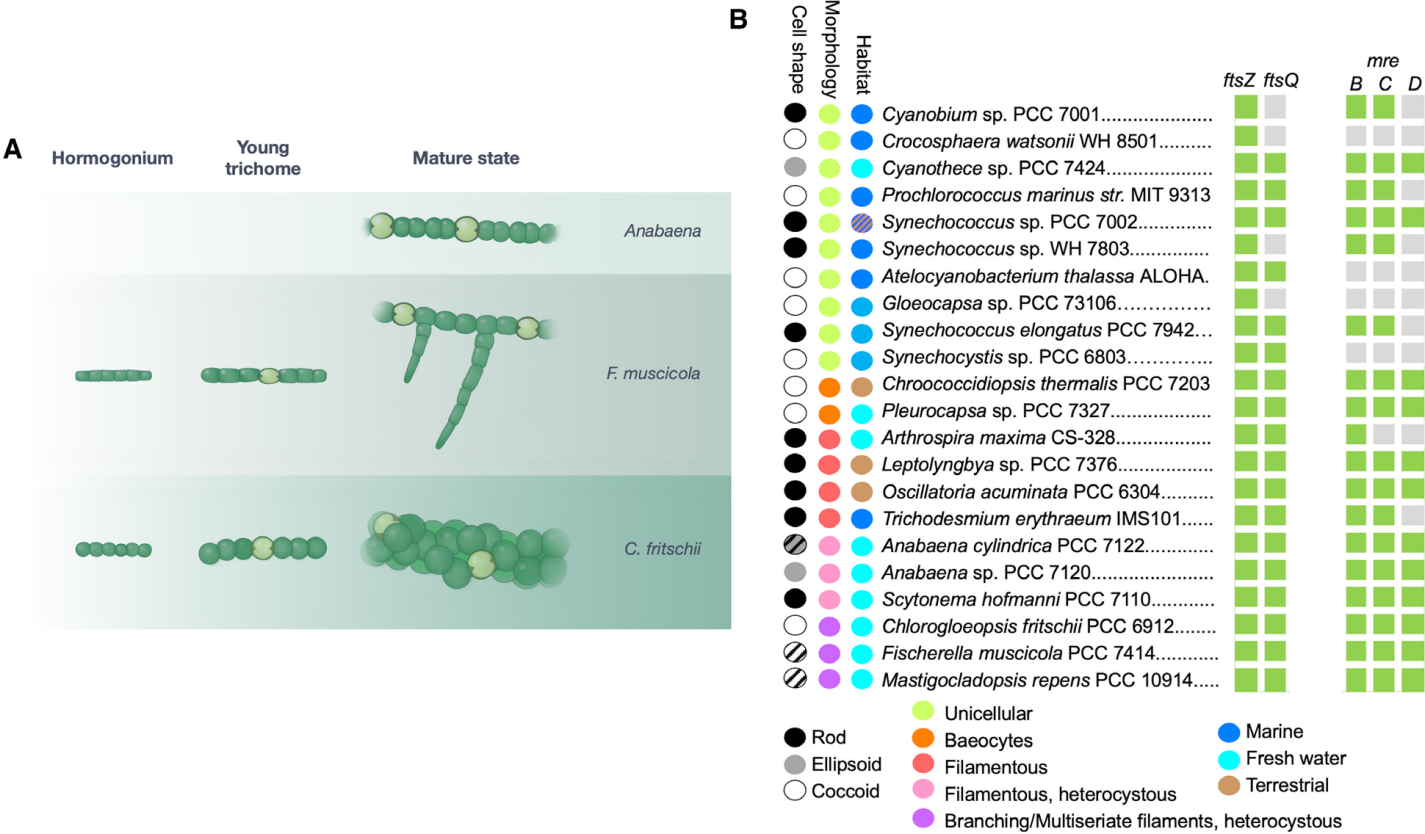
Presence of *ftsZ* and *mreB* homologs in cyanobacteria. (A) Illustration depicting the cellular morphologies and life stages of*F. muscicola*, *C. fritschii*, and *Anabaena* wild‐type trichomes. (B) Depiction of representative cyanobacterial species with different cell shapes and morphologies and from different habitats. The complete dataset is shown in File S1. Species, where homologous genes to *ftsZ*, *ftsQ*, *mreB*, *mreC*, and *mreD* were detected, are marked by a green square. Organisms, where a homolog was not found, are marked by a gray square.

## Results

### Conservation and distribution of *mreB* and *ftsZ* genes in cyanobacteria

To gain an insight into the sequence conservation of cyanobacterial MreB and FtsZ proteins, we performed a comparative sequence analysis of 131 cyanobacterial genomes, including representative strains for the various cyanobacterial cell morphologies. Our analysis reveals that, except for four cyanobacterial genomes, *mreB* is present in all tested cyanobacteria and its protein sequence is conserved (52% identical amino acids of all MreB proteins from all tested cyanobacterial species). Notably, spherical‐shaped *Prochlorococcus* spp. contain *mreB* and *mreC* homologs but lack *mreD* homologs, suggesting that the lack of *mreD* is sufficient to prevent rod‐shape formation in these species (File S1). This tempting hypothesis could be addressed in future studies. All of the four cyanobacterial species that lack *mreB* are unicellular cocci (Fig. 1B), which is in accordance with the rod shape‐determining function of *mreB*. In addition, few unicellular cyanobacteria and the linear filament forming *Trichodesmium* contain a partial *mre* operon that lacks *mreD*, while the filamentous *Arthrospira maxima* CS‐328 contains only *mreB* and lacks *mreCD* (Fig. 1B; File S1). The FtsZ encoding gene is present in all genomes from the dataset and its protein sequence is highly conserved (70% identical amino acids of all FtsZ proteins from all tested cyanobacterial species). In cyanobacteria, *ftsZ* is commonly encoded in close proximity to *ftsQ*, whose translated protein is a part of the divisome machinery, except for strains of the marine pico‐cyanobacteria Syn‐Pro‐Cya clade where *ftsQ* homologs are absent (Fig. 1B; File S1). Notably, *ftsA*, which is commonly found adjacent to *ftsZ* and *ftsQ* in the chromosome [41], is absent in all tested cyanobacteria as was previously shown for a smaller subset of cyanobacteria [27, 33].

### PG biosynthesis staining reveals alternative modes of cell growth in multicellular cyanobacteria

**Fig. 2:**
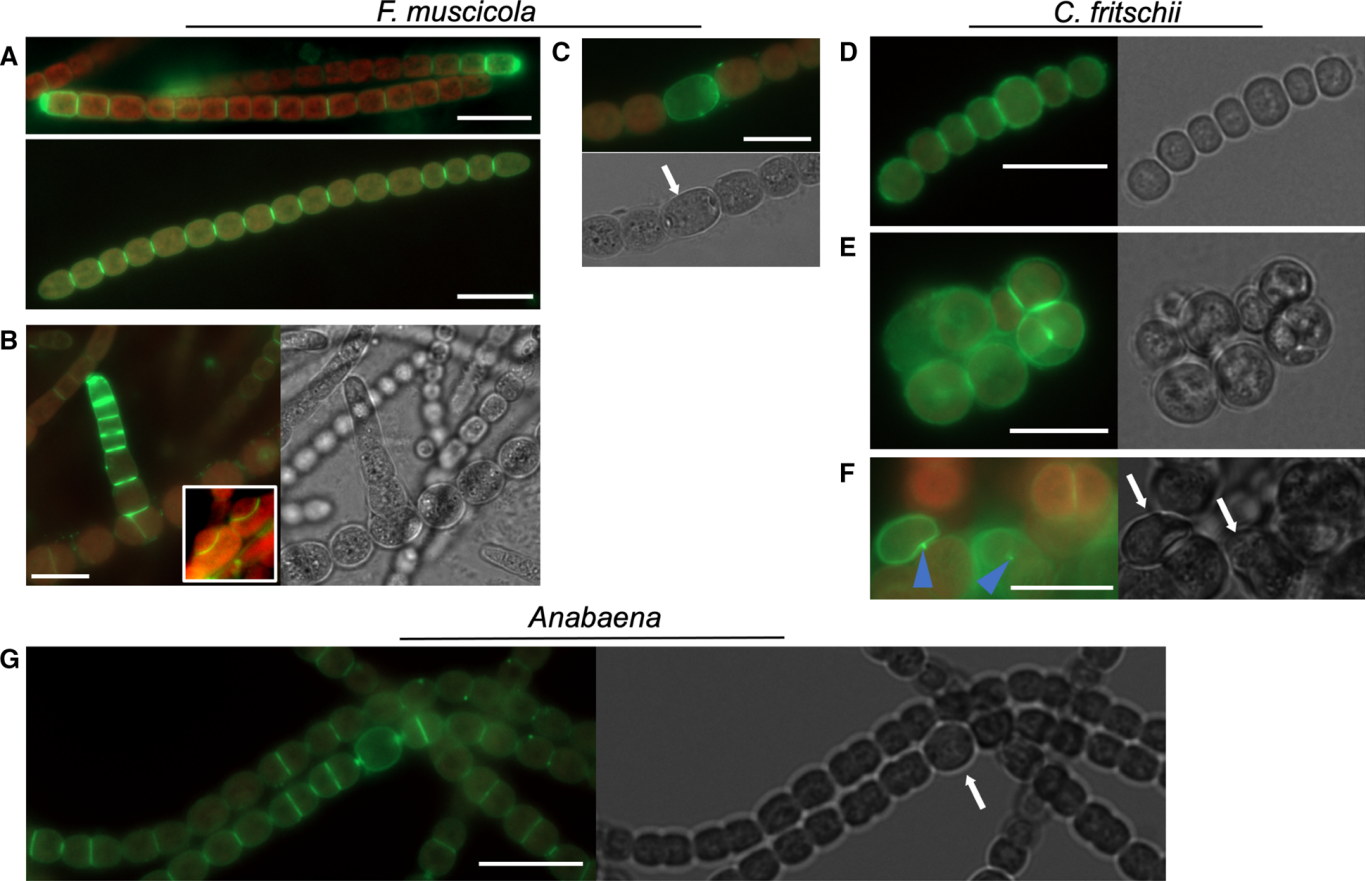
Fluorescent vancomycin labeling reveals different growth modes in multicellular cyanobacteria. (A–G) Bright‐field and merged chlorophyll autofluorescence (red) and BODIPY ^®^FL Vancomycin (Van‐FL) fluorescence micrographs of (A–C) *F. muscicola*, (D–F)*C. fritschii*, and (G) *Anabaena* cells stained with 5 µg·mL^−1^Van‐FL. Micrographs indicate different growth stages of the respective cyanobacterium: (A upper image) hormogonia, (A lower image) hormogonia in the transition phase to young linear trichome. Apical staining was observed in 123 apical cells (39%) while 191 showed septal staining only (61%),*n* = 314. (B) young branches, (C, F) heterocysts, (D) hormogonium (as indicated by the linear growth mode, which is restricted to the early growth stages (i.e. hormogonia) of*C. fritschii*), (E) multiseriate trichome, and (G) mature trichome. White arrows indicate heterocysts. (B) and (B inlay) additionally show Van‐FL staining pattern at the branching points of newly formed lateral branches. Van‐FL staining patterns were consistent among three independent experiments. Scale bars: 10 µm.

To study the localization of the Stigonematalean cell division machinery, we first aimed to determine the pattern of cell wall biogenesis (*i.e*.*,* PG biogenesis) in the different morphotypes that represent the life cycle stages of *F. muscicola* and *C. fritschii* [21]. For this purpose, we visualized the sites of active cell growth in *F. muscicola*, *C. fritschii*, and *Anabaena* with a fluorescently labeled vancomycin derivative (Van‐FL) and the fluorescent d‐amino acid HADA at different growth stages. Note that control cultures not supplemented with Van‐FL did not show any staining pattern for any of the three investigated species (Fig. S1). For the fast‐growing hormogonia from *F. muscicola*, we observed two different labeling phenotypes. Van‐FL either accumulated at the polar tips and the lateral walls from cylindrical apical cells, which are typically located at the tip of hormogonia [42] (39% of counted hormogonia; Fig. 2A upper image), while in hormogonia that are in a transition phase to young trichomes (indicated by pointed or rounded apical cells of the hormogonia; [42]), only the septal cell wall was stained by Van‐FL (61% of counted trichomes; Fig. 2A lower image). In mature trichomes (composed of wide cells; [21, 42]), cells at the tip of a newly formed lateral branch were characterized by an intense Van‐FL staining pattern that stained the polar tip as well as the lateral and septal cell wall of the fresh branches (Fig. 2B), which is in accordance with a rapid growth at branching points. This observation is similar to the pattern observed for hormogonia (Fig. 2A) and in concert with previous descriptions of structural similarities between hormogonia and young branches [42]. In contrast, staining of the main branch in mature *F. muscicola* trichomes revealed exclusively septal Van‐FL staining pattern (Fig. 2B). These observations indicate that polar/tip growth is exclusively restricted to rapidly growing cells in *F. muscicola* and that septal growth dominates in older trichomes. We also observed that cells right at the branching point still displayed septal staining; hence, these cells are still able to divide even after the branch is formed (Fig. 2B inlay). This observation is in contrast to the reported cell division dynamics in *Mastigocladus laminosus*, where cells in a branching point cease to divide after branching [43]. We further observed a notably strong Van‐FL staining of heterocysts (Fig. 2C), which is similar to what was previously reported for HADA (a fluorescent d‐amino acid; [44]) and Van‐FL‐stained *Anabaena* [45, 46]. HADA staining of *F. muscicola* further confirmed a predominant labeling of heterocysts but was otherwise found to only stain the septal cell wall, regardless of the growth stage (Fig. S2). Fluorescent d‐amino acids like HADA specifically stain PG transpeptidase activity [47], while Van‐FL primarily binds to exposed lipid II [9] and thus labels sites of active transglycosylation [48]. The lack of HADA staining at the apical sites of hormogonia could indicate that transglycosylation primarily takes place at those sites, suggesting that transglycosylation and transpeptidation could be spatially separated in *F. muscicola*. Considering that hormogonia are fast‐growing cell types [42], this observation potentially indicates that flexible PG strands could first be polymerized at the apical sites and then be rigidified by cross‐linking at other sites of the cell, including the septum.

The Van‐FL staining of *C. fritschii* shows a dim but distinguishable homogenous accumulation of Van‐FL in the membrane and a pronounced septal staining of *C. fritschii* young hormogonia (Fig. 2D), mature multiseriate trichomes, and aseriate aggregations (Fig. 2E,F). This observation is consistent with continuous growth and cell division as we never observed a clear pattern of the growth direction (Fig. 2D–F). Similar to *F. muscicola*, heterocysts displayed a distinct staining pattern, with a specific polar accumulation of Van‐FL signal at the sites of cell contact to the neighboring cell (Fig. 2F). In contrast, in *Anabaena*, Van‐FL staining was always septal and never observed at the lateral cell wall, except for a dominant staining of the whole heterocyst cell envelope (Fig. 2G). Like in *F. muscicola*, HADA only stained the septal cell wall as well as heterocysts in *C. fritschii* and *Anabaena* (Fig. S2). These different observations of cell wall biogenesis in the three different species suggest an intergeneric diversity of growth modes in multicellular cyanobacteria.

### Cellular localization of MreB

**Fig. 3:**
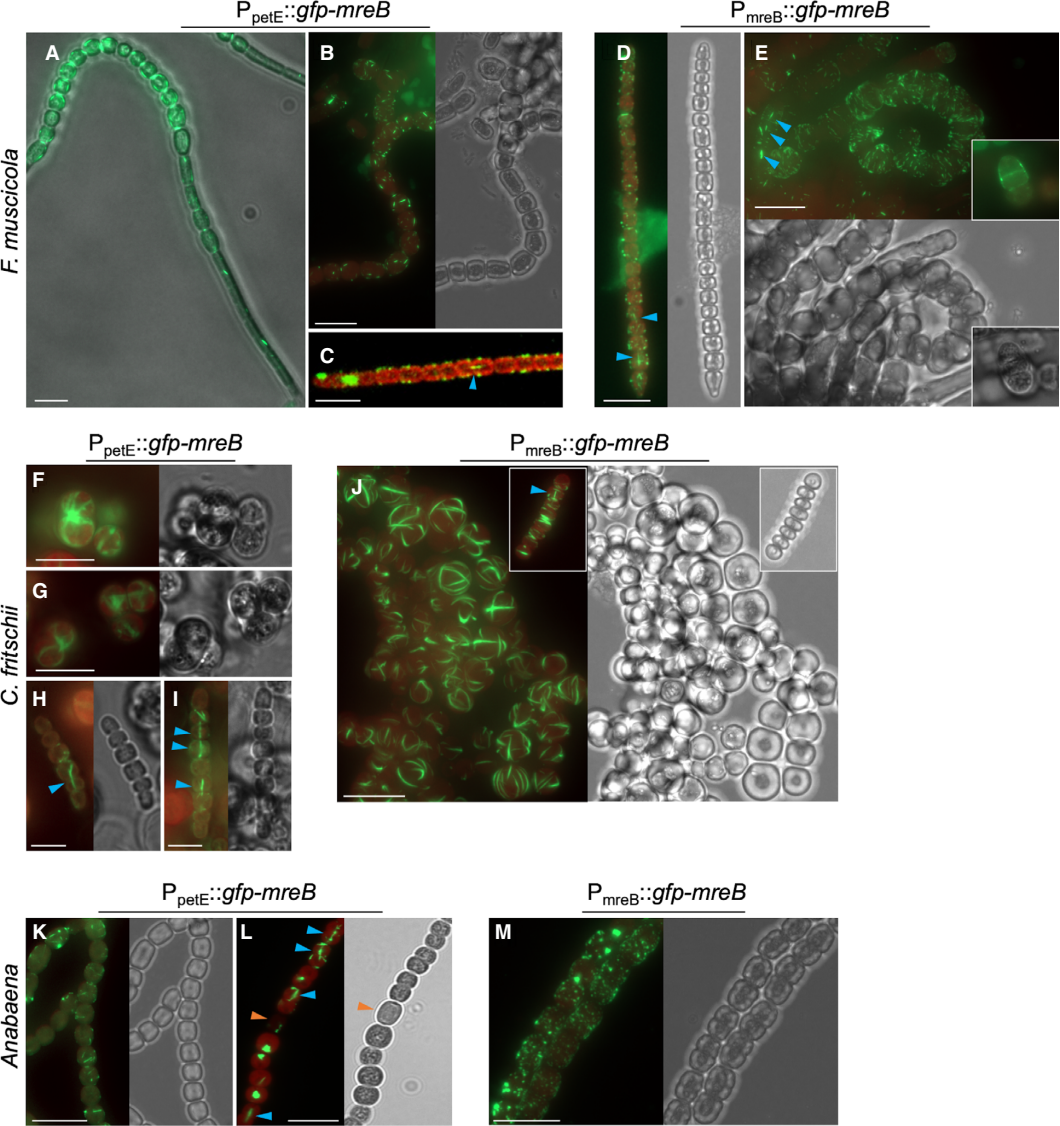
Localization of GFP‐MreB in *F. muscicola*, *C. fritschii*, and *Anabaena*. Bright‐field and merged chlorophyll autofluorescence (red) and GFP fluorescence micrographs of (A–E) *F. muscicola*, (F–J) *C. fritschii*, or (K–M) *Anabaena* expressing *gfp‐mreB* from (A–C, F–I, K–L) P_petE_ or from (D, E, J, M) P_mreB_. Figures show (A) mature trichome with nascent hormogonium, (B, E–G, J, K–M) mature trichomes and (D, H, I, J inlay) hormogonia. A heterocyst is marked with an orange triangle. Blue triangles indicate longitudinal GFP‐MreB filaments that appear to traverse the cells along the growth axis. Note: *Anabaena*expressing *gfp‐mreB*from P_petE_shows several small filaments throughout the cells when grown on BG11 medium, while polar GFP‐MreB plugs are only observed upon transfer to BG11_0_(BG11 without combined nitrogen), which we found to seemingly increase P_petE_‐driven expression, thus indicating that the GFP spots seen in (L) could be inclusion body‐like aggregates. Scale bars: (A–G, J–M) 10 µm or (H, I) 5 µm.

Likely as a result of their sophisticated endonuclease array and thick exopolysaccharide layer [35], genetic modification, including gene deletions or chromosomal insertions in Stigonematales cyanobacteria, is currently not available, obstructing the investigation of native protein localization, functionality of fusion proteins, and gene functions through gene deletions. Nonetheless, other heterologous options for protein localization studies are available and have successfully been used in *F. muscicola* and *C. fritschii* [36]. Thus, to investigate the localization of MreB in the three different morphotypes, we ectopically expressed translational *gfp‐mreB* fusions under the control of their native promoters (P_mreB_) and the copper inducible *petE* promoter from *Anabaena* (P_petE_) from the replicative pRL25C plasmid. The pRL25C plasmid, together with P_petE,_ has previously been employed to visualize MreB and FtsZ in *Anabaena* [24, 28, 29] and as such are suitable for the localization of both proteins in filamentous cyanobacteria. GFP localization in control experiments of *F. muscicola*, *C. fritschii*, and *Anabaena* expressing *gfp* from P_petE_ alone was cytoplasmic and never showed any discernible structures *in vivo* (Fig. S3). Notably, overexpression of *gfp*‐*mreB* did not cause any observable morphology alterations in any of the three cyanobacteria tested, regardless of the employed promoter (Fig. 3). This is in contrast to the swollen cell phenotype in an *Anabaena gfp‐mreBCD* overexpression strain [24], suggesting that overexpression of the whole *mre* operon and not just *mreB* is responsible for this cell morphology change. Filamentous strings of various lengths of GFP‐MreB from *F. muscicola* (termed GFP‐MreB_Fm_) were visible in all *F. muscicola* growth stages and cell types, including nascent hormogonia (which typically extend from the tips of the main trichome or branches and are later detached; Fig. 3A), mature trichomes (Fig. 3B,E) and hormogonia/young trichomes (Fig. 3C,D). However, abundance of GFP‐MreB_Fm_ filaments was seemingly decreased in nascent hormogonia compared to other cells of the trichome (Fig. 3A). GFP‐MreB_Fm_ typically formed short filamentous strings throughout the cells, without any directional preference (95% cells when expressed from P_mreB_, *n* = 5875; see File S2 for a detailed description of all MreB quantification analysis), except in some cells where we saw linear GFP‐MreB_Fm_ filaments aligned at the lateral cell axis (Fig. 3C,D,E, 1.3% cells when expressed from P_mreB_, *n* = 5875) that sometimes seemed to cross cell–cell borders (Fig. 3D). Septal localization of GFP‐MreB_Fm_ was also observed in all cell types albeit in a low proportion of cells (5.6% cells when expressed from P_mreB_, *n* = 5875); septal localization increased when GFP‐MreB_Fm_ was expressed from the *petE* promoter (12.5%, *n* = 606 cells). Rarely, we also observed that GFP‐MreB_Fm_ seemingly localized to the mid‐cell in fresh branching points (Fig. 3E inlay). Because fluorescent tags may influence the MreB localization [49], we investigated the localization of MreB in addition by immunofluorescence of cells expressing polyhistidine tagged *mreB*_Fm_ from P_petE_. This confirmed the localization of GFP‐MreB_Fm_ and revealed that MreB_Fm_‐His also formed filamentous strings throughout the cells and occasionally localized to the mid‐cell in rings (Fig. S4A, 0.4%, *n* = 1064 cells). Immunostaining also confirmed the existence of MreB filaments longitudinal to the main long axis of the cells (Fig. S4B, 6.9%, *n* = 1064 cells). Consequently, we conclude that the GFP tag does not affect MreB localization, which is also in concert with a fully functional GFP‐MreB_Ana_ fusion in *Anabaena* [24]. Additionally, we observed diffuse and patchy nonfilamentous localization of MreB_Fm_‐His in young branches that were not present in control cells (Fig. S4C,D). The patchy localization of MreB_Fm_‐His directly correlates with the prominent Van‐FL staining in nascent hormogonia/young branches (Fig. 2A,B) and might also explain why we only rarely observed GFP‐MreB_Fm_ filaments in hormogonia.

In *C. fritschii*, GFP‐MreB (termed GFP‐MreB_Cf_) expressed from P_petE_ localized as comparably thick filamentous strings within the cells and showed a polar localization in dividing cells, where filaments raised and scattered from the pole in opposite directions of each dividing cell (Fig. 3F,G, 84.5% cells when expressed from P_petE_, *n* = 520), reminiscent of the spindle apparatus in eukaryotes [50]. Similar to what we observed in *F. muscicola*, longitudinal GFP‐MreB_Cf_ filaments were also observed in *C. fritschii*, although only in hormogonia, the only stage at which *C. fritschii* grows as trichomes (Fig. 3H,I, 5.2% cells when expressed from P_petE_, *n* = 520). Expression of *gfp‐mreB*_Cf_ from P_mreB_ resulted in an even more abundant and extensive network of thick filamentous strings (93.7%, *n* = 477 cells) but also revealed longitudinal GFP‐MreB_Cf_ filaments in hormogonia (Fig. 1, 3, 1%, *n* = 477 cells). In *Anabaena*, we observed both, GFP‐MreB (termed GFP‐MreB_Ana_) filament strings appearing as bundles that traversed throughout the cells within a trichome (Fig. 3K,L) but also longitudinal GFP‐MreB_Ana_ filaments (Fig. 3M) when expressed from P_petE_. Consistent with previous reports [24], GFP‐MreB_Ana_ also formed foci at the septa between neighboring cells (Fig. 3L, 14%, *n* = 636 cells) when grown in nitrogen‐deprived growth medium (i.e. BG11_0_), which we found to seemingly increase P_petE_‐driven expression (19%, *n* = 1676 cells). Thus, we speculate that the foci observed by [24] correspond to inclusion body‐like accumulations of GFP‐MreB_Ana_. When expressed from P_mreB_, GFP‐MreB_Ana_ revealed filamentous strings (86%, *n* = 636 cells), although those strings were considerably shorter and more abundant (Fig. 3M), resembling the native localization of MreB in *E. coli* (as shown with a MreB and RFP sandwich fusion, MreB‐RFP^SW^) [49]. In the context of our analysis on MreB localization, we observed that all three cyanobacterial native *mreB* promoters can also be employed by *E. coli* and lead to prominent GFP‐MreB filaments within the cells (Fig. S5A) that show motile properties (Video [Link], [Link], [Link]), suggesting a certain functionality of the generated GFP‐MreB fusion proteins. Taken together, MreB forms abundant filamentous strings within all three cyanobacterial species, the *mreB* promoter seemingly leads to the formation of more abundant GFP‐MreB filaments and GFP‐MreB_Cf_ unlike GFP‐MreB_Fm_ and GFP‐MreB_Ana_, had the tendency to form thick filamentous strings in *C. fritschii*.

In *Anabaena*, deletion of *mreB* was previously shown to affect cell shapes, leading to a rounded/swollen morphotype [24]. To gain further insights into the MreB function, we attempted to produce *mreB* deletion mutants in *F. muscicola* and *C. fritschii* using double homologous gene replacements. However, even despite the unusually long fragments used for homologous recombination (2–3 kbp upstream and downstream), we remained unsuccessful to generate any gene deletion or gene knockdown strains. Notably, in *Anabaena*, homologous flanks of around 600 bp or less are generally sufficient to allow homologous recombination [51, 52], indicating that homologous recombination efficiency is remarkably low in subsection V cyanobacteria.

**Fig. 4:**
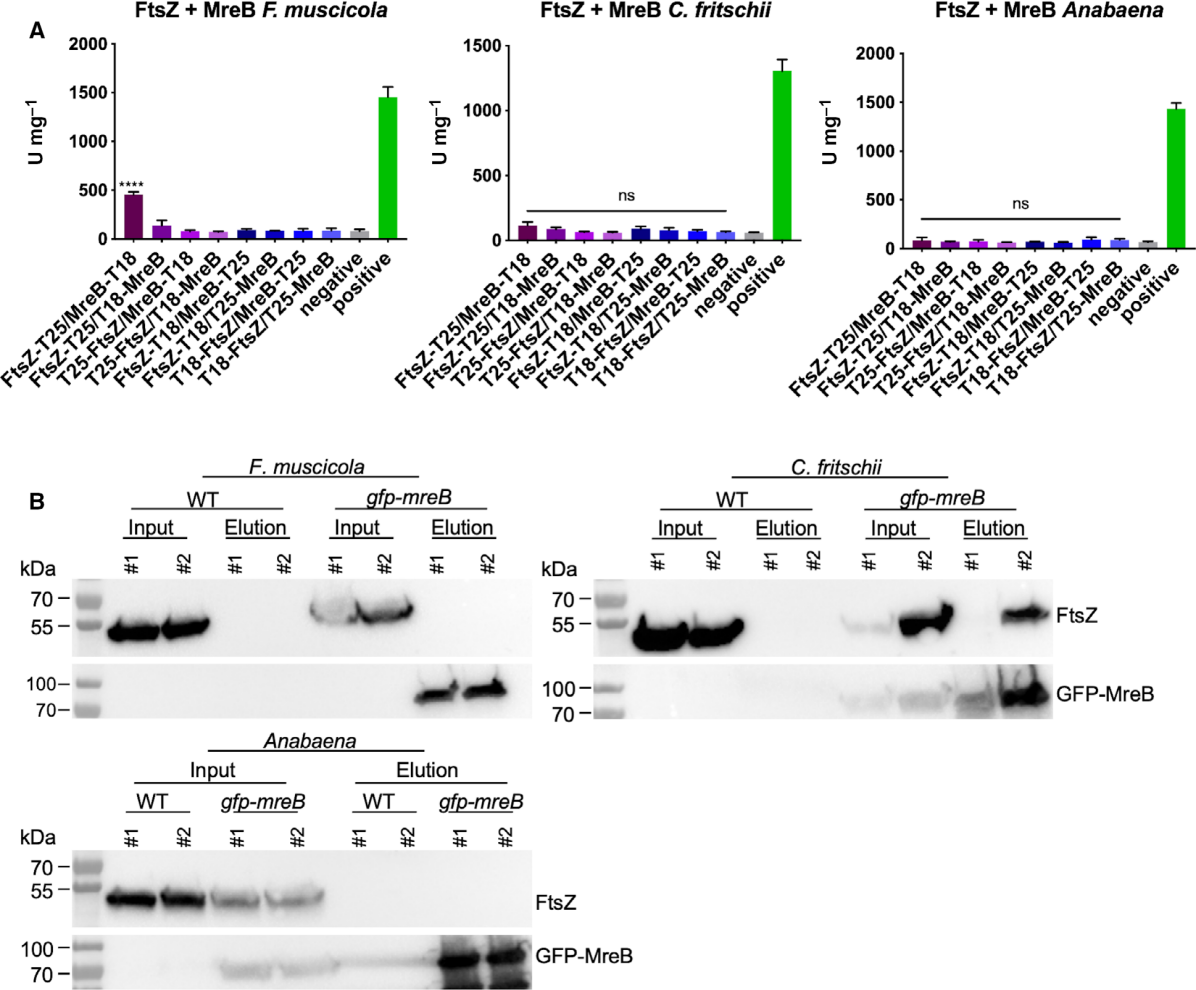
Interaction of FtsZ and MreB in multicellular cyanobacteria. (A) Beta‐galactosidase assays of*Escherichia coli*cells co‐expressing indicated translational fusion constructs of all possible pairwise combinations of*ftsZ*with*mreB*. Quantity values are given in Miller units per milligram LacZ of the mean results from three independent colonies. Error bars indicate standard deviations (*n* = 3). Neg: pKNT25 plasmid carrying*ftsZ*co‐transformed with empty pUT18C. Pos: Zip/Zip control. The value indicated with **** is significantly different from the negative control (****P < 0.0001; Dunnett's multiple comparison test and one‐way ANOVA). (B) Anti‐FtsZ_SubsV_and anti‐GFP western blots from anti‐GFP co‐IP's from two individual biological samples of*F. muscicola*,*C. fritschii*, and*Anabaena*cell‐free extracts expressing*gfp‐mreB*from P_mreB_or WT control cells. Experiments were performed twice, each time using two independent biological replicates for each sample type (i.e. WT and GFP‐tagged MreB expressing strain). Input samples were taken before addition of anti‐GFP antibodies.

Previous reports have shown that MreB interacts directly with FtsZ in *E. coli* [53]. In *F. muscicola* and *C. fritschii*, GFP‐MreB is not localized to the mid‐cell; nonetheless, we observed mid‐cell localization of MreB_Fm_‐His (Fig. S4A) and a localization of GFP‐MreB at the sites of cell–cell connections or forming septa in some morphotypes of *F. muscicola* (mature trichomes with wide cells in Fig. 3A, young branching point in Fig. 3E inlay), *C. fritschii* (dividing cells and aggregates in Fig. 3F,G) and in *Anabaena* trichomes (Fig. 3L,M). This, together with the potential connection of GFP‐MreB through contiguous cells within a trichome (Fig. 3C,H,I,L), tempted us to test for an interaction between MreB and FtsZ as well as between MreB and the septal‐localized protein SepJ [51, 54] by bacterial adenylate cyclase two‐hybrid (BACTH) assays and co‐immunoprecipitation (co‐IP). The BACTH assays showed that MreB and FtsZ only interacted in *F. muscicola* (Fig. 4A) and that none of the three MreB proteins interacted with SepJ (Fig. S6). In contrast, *in vivo* studies using anti‐GFP co‐immunoprecipitation analysis of cells expressing GFP‐MreB from P_mreB_ revealed an interaction between GFP‐MreB_Cf_ and FtsZ_Cf_ in *C. fritschii* (Fig. 4B). As such, it seems that MreB and FtsZ from *F. muscicola* and *C. fritschii* can interact with each other, but this interaction is possibly dependent on the protein tag that is attached to MreB.

### Overexpression of *ftsZ* leads to mid‐cell ring disappearance and aberrant morphologies

**Fig. 5:**
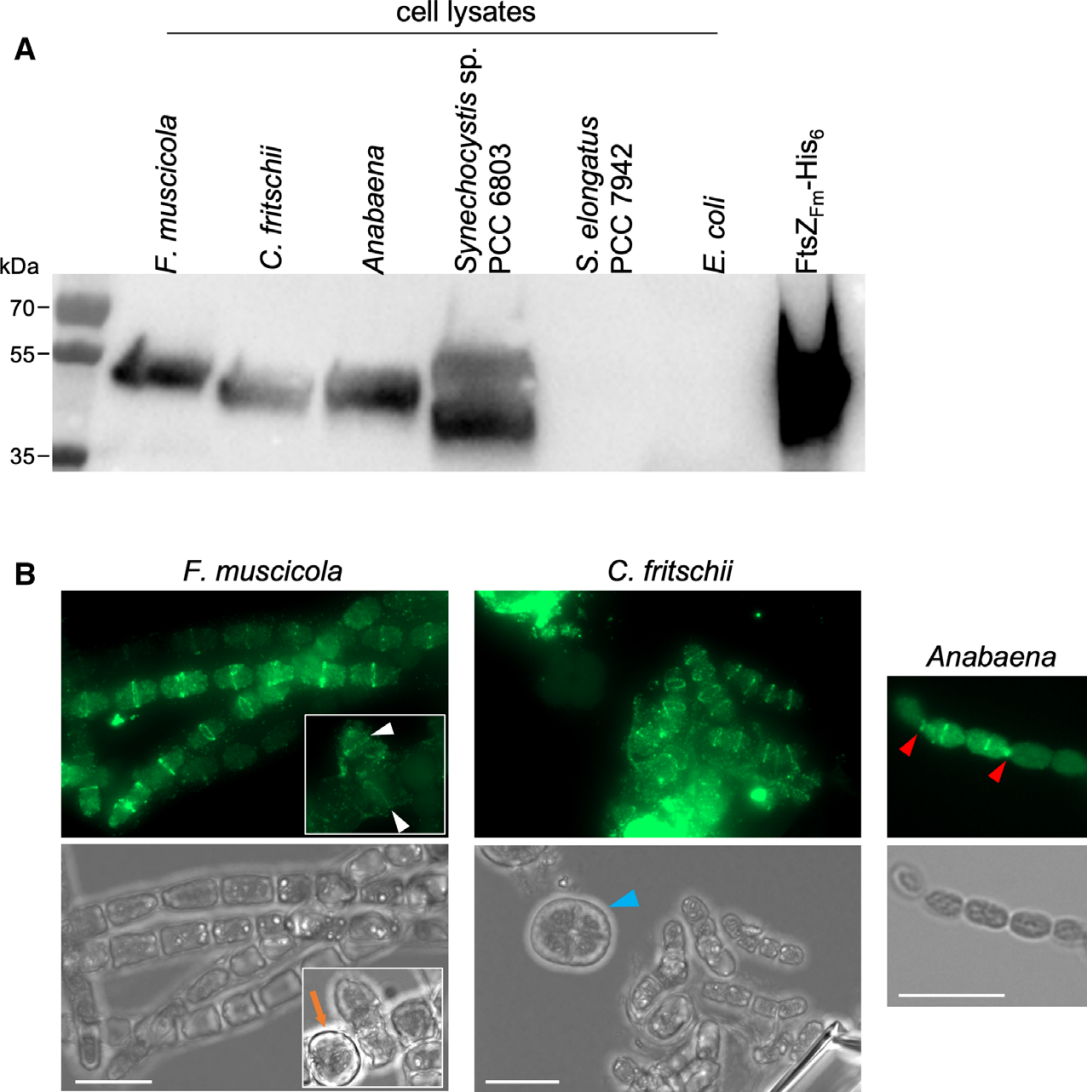
Assessment of new Anti‐FtsZ_SubsV_antibody. (A) Western blot analysis of cell‐free lysates from*F. muscicola*,*C. fritschii*,*Anabaena*,*Synechocystis*,*Synechococcus*, and*Escherichia coli*using anti‐FtsZ_SubsV_antibody (raised against*F. muscicola*and*C. fritschii*FtsZ). As a control, 1 µg purified FtsZ_Fm_‐His was included. (B) Immunolocalization of FtsZ in*F. muscicola*,*C. fritschii*, and*Anabaena*using an anti‐FtsZ_SubsV_antibody. White triangles show Z‐rings in branching cells. The orange arrow indicates a heterocyst. The blue triangle points to a mature*C. fritschii*cell which was not permeabilized. Red triangles indicate septal localization of FtsZ in*Anabaena*. Results shown in here are representative figures from three independent experiments. Scale bars: 10 µm.

Multiplanar cell division in Stigonematales likely requires reorganization of the cell division machinery. This type of cell division occurs in mature trichomes at later stages of the cell cycle, where newly formed branches are randomly placed along the trichomes [21]. To first examine the Z‐ring placements within Stigonematales in more detail, we visualized FtsZ ring formation in different growth stages of *F. muscicola*, *C. fritschii*, and *Anabaena* using a newly generated polyclonal anti‐FtsZ antibody. While it was initially designed to detect specifically *F. muscicola* and *C. fritschii* FtsZ (see Materials and methods), we also found that it can detect *Anabaena* and *Synechocystis* FtsZ but fails to identify *S. elongatus* PCC 7942 and *E. coli* FtsZ (Fig. 5A). Our results show that mid‐cell FtsZ rings are present in most young and older trichomes but were never observed in *F. muscicola* heterocysts (Fig. 5B inlay), which is in accordance with the absence of Z‐rings in *Anabaena* heterocysts [29]. The here developed immunofluorescence protocol, which accounts for the intensive cell envelopes of subsection V cyanobacteria [35], also allowed us to visualize the presence of FtsZ rings in branching points of *F. muscicola*, which we never saw using the protocol previously developed for *Anabaena* [31]. However, we could not visualize any Z‐rings in mature *C. fritschii* cells (Fig. 5B) and Z‐rings in *C. fritschii* could only be detected in hormogonia (Fig. 5B), suggesting that the thick cell envelope of mature *C. fritschii* cells cannot be penetrated even when using our extensive permeabilization procedures.

Overexpression of *ftsZ* or impairment of proper FtsZ function has been shown to induce cell filamentation in *E. coli* [55] and *Synechococcus* [27, 56], respectively. Thus, we initially investigated whether overproduction of the three different FtsZ proteins from *F. muscicola*, *C. fritschii*, and *Anabaena* resulted in the same filamentous phenotype in *E. coli* and observed similar cell filamentations for all three proteins (Fig. S5B). Unlike *gfp‐mreb*, which could be expressed in *E. coli* from the cyanobacterial native *mreB* promoters, *E. coli* cells were not found to be able to utilize the respective native cyanobacterial *ftsZ* promoters (Fig. S5C). Using a similar approach as [20], we also verified that all three FtsZ proteins were able to form comparable protein polymers *in vitro* (Fig. S5D). To test the sensitivity of *F. muscicola*, *C. fritschii*, and *Anabaena* to increased FtsZ dosage, we created C‐ and N‐ terminal translational fusions of FtsZ with GFP. These were expressed from pRL25C from three different promoters including the respective native promoters (P_ftsZ_), P_petE_ (promoter sequence from *Anabaena*, can be utilized by all three tested cyanobacteria) and the nitrogen inducible P_glnA_ (sequence from *C. fritschii*, which we found works in all three tested cyanobacteria). Unlike in *E. coli*, ectopic overexpression of *ftsZ‐gfp* from P_petE_ on the replicative pRL25C plasmid neither impaired cell division nor Z‐ring formation in *Anabaena*.

**Fig. 6:**
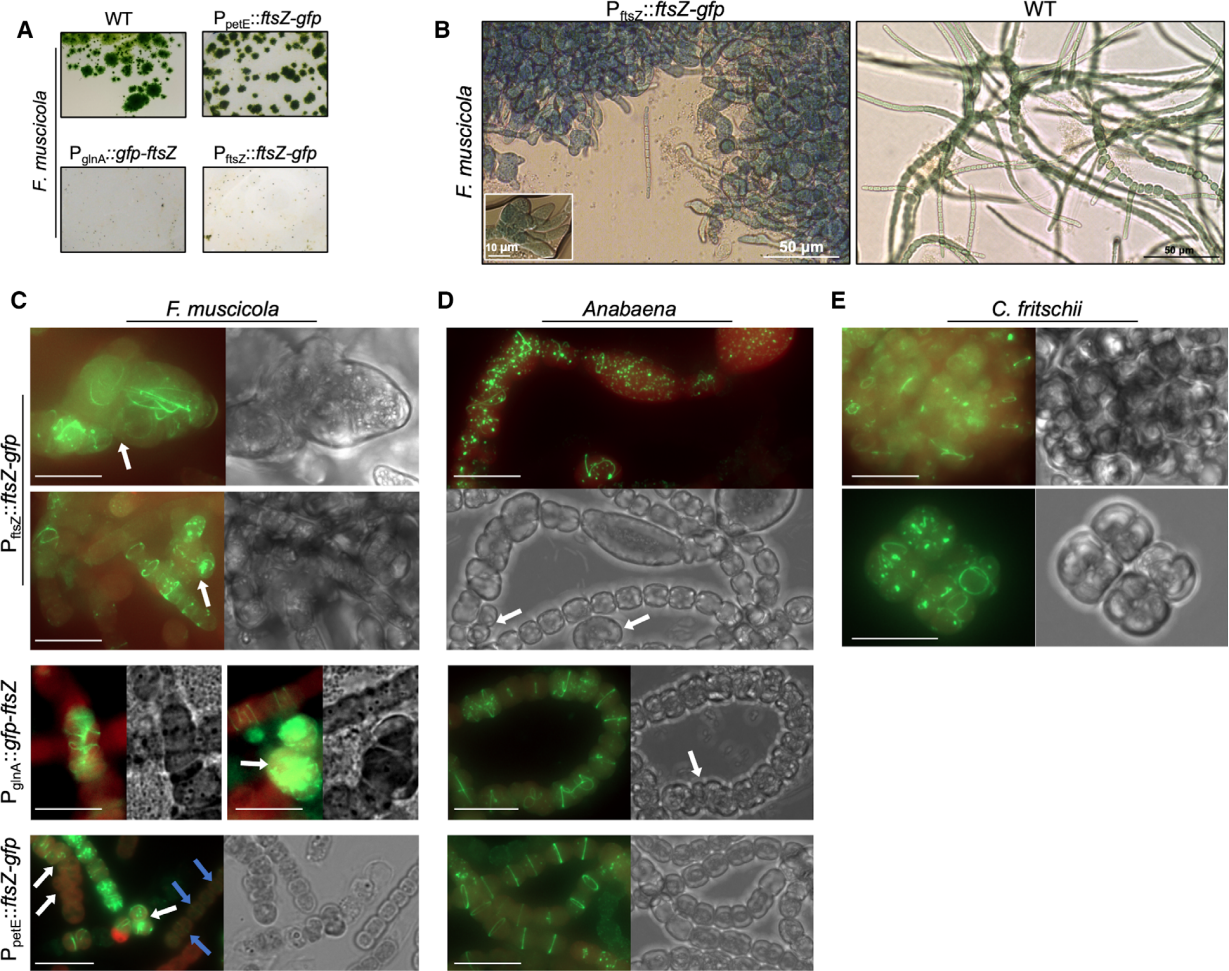
*Fischerella muscicola*and*Anabaena*are sensitive to excess FtsZ levels. (A) Micrographs of mating filters showing growth of*F. muscicola*WT colonies and*F. muscicola*colonies after transformation with pRL25C plasmids carrying P_petE_::*ftsZ‐gfp*, P_glnA_::*gfp‐ftsZ*or P_ftsZ_::*ftsZ‐gfp*. (B) Bright‐field micrograph showing morphological alterations (i.e. swelling) of*F. muscicola*cells expressing*ftsZ‐gfp*from P_ftsZ_. (C–E) Merged chlorophyll autofluorescence (red) and GFP fluorescence and bright‐field micrographs of (C)*F. muscicola*, (D)*Anabaena*or (E)*C. fritschii*expressing*ftsZ‐gfp*or*gfp‐ftsZ*from P_ftsZ_, P_glnA_or P_petE_. P_petE_‐driven expression of*ftsZ‐gfp*in*F. muscicola*and*Anabaena*was additionally increased using 0.2 µmCuSO_4_. White arrows indicate trichomes with multiseriate trichome growth. Blue arrows mark dim Z‐ring formations. Scale bars: (C–E) 10 µm.

Branch formation in Stigonematales cyanobacteria is likely the result of coordinated positioning of the divisome in branching points [40, 43]. We thus hypothesized that different FtsZ levels could impact divisome function and consequently coordinated branch formation in Stigonematales. To test for this, we overexpressed *ftsZ* from three different promoters: P_petE_, the native P_ftsZ_ promoter, and P_glnA_ (from *C. fritschii*), which is known to be constitutive in *F. muscicola* and *C. fritschii* in nitrate containing growth medium [36]. *F. muscicola* cells expressing *ftsZ_Fm_‐gfp* from P_petE_ grew as *F. muscicola* wild‐type (WT) on solid media (containing 0.2 µm copper by default), whereas expression driven by P_glnA_ and P_ftsZ_ considerably impaired colony and cell growth (Fig. 6A–C), as indicated by the tiny colonies and the strains disability to spread across the filter (Fig. 6A). In accordance with this, motile *F. muscicola* hormogonia were seldomly seen; hence, impaired motility is an indirect consequence of *ftsZ‐gfp* overexpression (Fig. 6B). In liquid BG11, some *F. muscicola* cells expressing *ftsZ*_Fm_‐*gfp* from P_petE_ divided at different angles to the normal growth planes, forming multiseriate trichomes (Fig. 6C). FtsZ_Fm_‐GFP filaments were often tangled‐up, and Z‐rings were only seldomly observed within the cells (Fig. 6C). In contrast, clear mid‐cell rings were visible in *Anabaena* expressing *ftsZ*_Ana_‐*gfp* from P_petE_ and no multiseriate trichomes or swollen cells were observed (Fig. 6D). Expressing *ftsZ*_Fm_‐*gfp* from P_ftsZ_ led to the formation of swollen cells in *F. muscicola* and *Anabaena*. Swollen *F. muscicola* cells never displayed FtsZ_Fm_‐GFP rings; instead, FtsZ_Fm_‐GFP filaments appeared longitudinal to the main trichome axis or spread throughout the cells (Fig. 6C). Clear rings were only present in cells with WT‐like phenotype (Fig. 6C). Some *F. muscicola* and *Anabaena* cells expressing *ftsZ* from P_ftsZ_ underwent multiple divisions in all planes (Fig. 6C,D), indicating that indeed the levels of FtsZ can influence multiplanar cell division.

No apparent morphological changes were present in *C. fritschii* cells expressing *ftsZ*_Cf_‐*gfp* from P_ftsZ_ (Fig. 6E). However, as *C. fritschii* WT already grows in multiseriate trichomes with individual cells showing diverse morphotypes, a precise assertion of morphogenic and cell division properties of overexpressed *ftsZ*_Cf_‐*gfp* is only limited. Besides normal Z‐rings, we also observed multiple FtsZ_Cf_‐GFP rings throughout the cells (Fig. 6E), indicating that overexpression of *ftsZ*_Cf_‐*gfp* partially affects Z‐ring formation and placement in *C. fritschii*. Overexpression of *gfp‐ftsZ*_Fm_ from P_glnA_ in *F. muscicola* resulted in multiseriate trichomes, where multiple divisions lead to small cells that appeared compressed within the trichome as well as accumulations of GFP‐FtsZ_Fm_ filaments in those cells (Fig. 6C, left panel). Nonetheless, trichomes with WT‐like morphology could still be observed in the culture and those cells revealed the formation of multiple contiguous GFP‐FtsZ_Fm_ rings (Fig. 6C, right panel). *Anabaena* expressing *gfp‐ftsZ*_Ana_ from P_glnA_ showed normal colony morphology on growth plates and GFP‐FtsZ_Ana_ mid‐cell rings as well as longitudinal and tangled‐up GFP‐FtsZ_Ana_ filaments could be observed (Fig. 6D). To some degree, multiseriate *Anabaena* trichomes were also detected upon overexpression of *gfp‐ftsZ*_Ana_ from P_glnA_ (Fig. 6D, Fig. S7), suggesting that the linear *Anabaena* trichome is also sensitive to elevated FtsZ levels. Neither *F. muscicola* nor *Anabaena* cells expressing *ftsZ‐gfp* or *gfp‐ftsZ* from P_ftsZ_ or P_gnlA_, respectively, were able to grow in liquid culture, obstructing any comparative analyses of intracellular FtsZ protein levels. We speculate that the assumed high levels of FtsZ in those strains are the cause for these growth defects. This notion is supported by the aberrant cell morphologies and the strongly inhibited colony growth in strains expressing *ftsZ* from P_ftsZ_ and P_glnA_ (Fig. 6A,C,D). In the course of our investigation, we also obtained a clone that had a point mutation at position 280 in the FtsZ_Ana_ coding sequence, exchanging the isoleucine with a phenylalanine (termed FtsZ^I280F^). The I280F mutation rendered *gfp*‐*ftsZ*_Ana_ (expressed from P_glnA_) nonfilament‐forming but still induced a multiseriate trichome growth in *Anabaena* (Fig. S7), suggesting that rather FtsZ abundance than precise Z‐ring placement dictates the linear growth pattern in *Anabaena*. We note that none of the branches that occurred in *Anabaena* overexpressing *ftsZ* ^I280F^_Ana_ led to the formation of true branches.

Attempts to express *ftsZ*_Cf_‐*gfp* or *gfp*‐*ftsZ*_Cf_ from P_petE_ and P_glnA_, respectively, remained unsuccessful as we never obtained any successfully transformed *C. fritschii* clones. Our results so far indicate that *F. muscicola* is more sensitive to increased FtsZ abundance in comparison to *Anabaena* and that different intracellular levels of FtsZ dictate the linear or multiseriate trichome phenotypes in filamentous cyanobacteria. This raises the possibility that multiplanar division in *F. muscicola* is more tightly controlled at the divisome level and is dependent on the FtsZ abundance within the cell.

### FtsZ in multicellular cyanobacteria is degraded *in vitro* by proteolysis

**Fig. 7:**
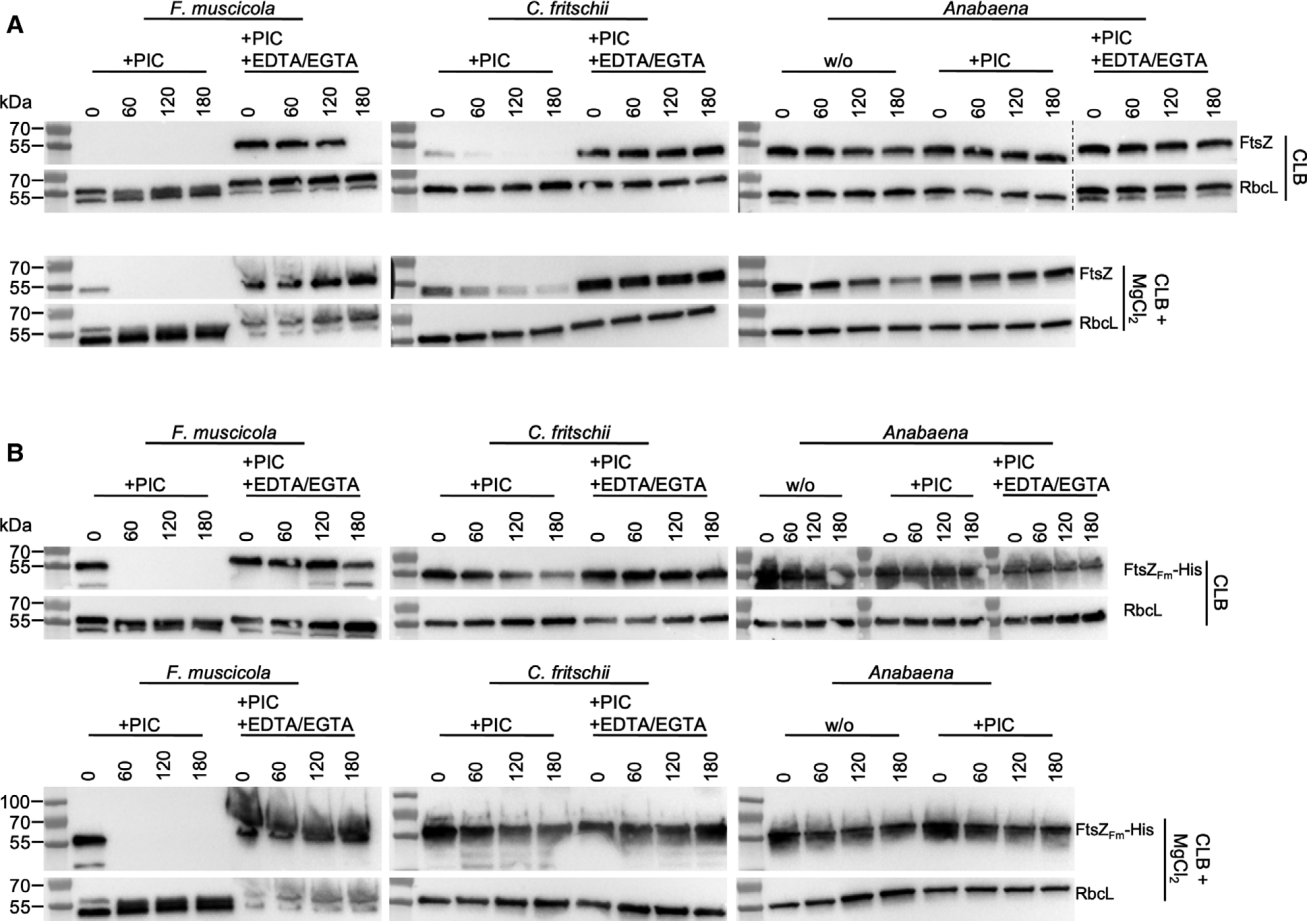
High rate of proteolytic degradation of FtsZ in subsection V cyanobacteria. (A) Anti‐FtsZ_SubsV_western blots of cell‐free extracts of*F. muscicola*,*C. fritschii*, and*Anabaena*incubated at 37 °C (*F. muscicola*and*C. fritschii*) or 30 °C (*Anabaena*) for 0, 60, 120, or 180 min supplemented or not (w/o) with protease inhibitor cocktails (PIC) and EDTA/EGTA. Cells were either lysed in cyanobacterial lysis buffer (CLB) or in CLB supplemented with 10 mmMgCl_2_. EDTA, and EGTA were supplemented as 5 mmeach, except in cases of presence of 10 mmMgCl_2_, where 7.5 mmEDTA and EGTA were included. (B) Purified FtsZ_Fm_‐His (1 µg per sampling time point) was incubated for the indicated time points in cell‐free extracts of*F. muscicola*,*C. fritschii*, or*Anabaena*supplemented or not (w/o) with PIC and EDTA/EGTA. Degradation of FtsZ_Fm_‐His was detected using an anti‐His antibody. (A, B) As a loading and specific‐degradation control, levels of RbcL were detected using an anti‐RbcL antibody. Images are representative western blots from duplicate experiments.

Intracellular protein level of FtsZ is regulated by proteolytic activity toward FtsZ in *Anabaena* [57], and we have often observed the complete absence of FtsZ in total protein extracts from *F. muscicola* and *C. fritschii* shortly after cell lysis. Consequently, to test if FtsZ is degraded by an intracellular protease in *F. muscicola* and *C. fritschii*, we incubated total protein extracts of *F. muscicola* and *C. fritschii* at 37 °C and *Anabaena* at 30 °C (the temperature was chosen according to the optimal growth temperatures of the strains) and supplemented the reactions either with a protease inhibitor cocktail (PIC), protecting against serine and cysteine proteases, with PIC and EDTA/EGTA (protecting additionally against metalloproteases) or without protease inhibitors. FtsZ levels were then detected by anti‐FtsZ_Fm_ immunoblotting. In our standard cyanobacterial lysis buffer (CLB) and even in the presence of serine and cysteine inhibitors (i.e. with PIC), *F. muscicola* FtsZ was found to be heavily subjected to proteolytic degradation as it was already degraded during the cell lysis and removal of cell debris steps (i.e. no FtsZ signal at T_0_; Fig. 7A). This proteolytic activity could only be mitigated upon supplementation of EDTA/EGTA (Fig. 7A), suggesting that FtsZ is degraded by a metalloprotease in *F. muscicola*. Similarly, although less prominent, FtsZ was also subject to proteolytic degradation in *C. fritschii* in the presence of PIC, while degradation could be terminated upon supplementation of EDTA/EGTA (Fig. 7A). In contrast, we did not observe any proteolytic activity against FtsZ in *Anabaena*, even when PICs were omitted (Fig. 7A). FtsZ has been shown to be proteolytically degraded by the ClpXP protease in *E. coli* [20] and buffers used for ClpXP protease‐mediated proteolytic degradation generally contain MgCl_2_ as co‐factor [20, 58, 59]. Consequently, we tested whether the addition of MgCl_2_ altered proteolytic activity in cell‐free cyanobacterial lysates. Indeed, we observed that the addition of MgCl_2_ could now promote degradation of FtsZ in *Anabaena* (Fig. 7A lower panel) to a similar degree as previously reported [57], and this degradation was sensitive to the presence of serine and cysteine protease inhibitors in the lysis buffer. Thus, this could indicate that *Anabaena* FtsZ might be a target of the serine protease ClpXP. In contrast, addition of MgCl_2_ decreased proteolytic activity in *F. muscicola* and *C. fritschii* (Fig. 7A lower panel), suggesting that different proteolytic systems regulate FtsZ levels in *Anabaena* compared to *F. muscicola* and *C. fritschii*.

Next, we wanted to investigate whether the differences in the FtsZ amino acid sequences between the different species might affect the proteolytic degradation. Therefore, we incubated purified and His‐tagged FtsZ from *F. muscicola* (FtsZ_Fm_‐His) in the respective cell‐free lysates and analyzed its degradation pattern by anti‐FtsZ_Fm_ immunoblotting. As expected, we found that FtsZ_Fm_‐His is readily degraded in *F. muscicola* (also showing that the His‐tag does not interfere with proteolytic degradation; Fig. 7B). However, proteolytic activity against FtsZ_Fm_‐His was decreased in *C. fritschii* and absent in *Anabaena*, even upon supplementation with MgCl_2_ (Fig. 7B). Consequently, the specific amino acid sequences or tertiary structures of the FtsZ protein are important for efficient proteolytic degradation. At all times, levels of the large subunit of RuBisCO protein, RbcL, remained constant, demonstrating that the proteolytic activity is specific against FtsZ (although we note that we cannot exclude that the protease that targets FtsZ in multicellular cyanobacteria might have other targets as well). Therefore, the propensity of FtsZ from *F. muscicola* and *C. fritschii* to rapid proteolytic degradation could be indicative for a high FtsZ turnover rate *in vivo*.

## Discussion

If Stigonematalean cyanobacteria contain the same repertoire of cell division proteins as unicellular and filamentous cyanobacteria [60], then why do they present such morphological variations? Our study suggests that part of the answer to that question lies within the expression and posttranslational regulation of FtsZ in this group.

In *F. muscicola*, we identified two modes of cell wall growth, lateral elongation and apical (polar) growth in young branches and hormogonia (i.e. young cell types) while older trichomes grew by septal growth only. We also observed septal wall growth in *C. fritschii* and *Anabaena*, as previously observed in the filamentous heterocystous cyanobacteria *Anabaena* and *Nostoc punctiforme* [45, 61, 62, 63, 64]. In rod bacteria, septal growth is an indication of cell division [65], whereas in coccoid bacteria or ovococci, both cell division and cell wall extension occur at mid‐cell [66]. In polar (i.e. apical) growing bacteria such as Actinobacteria, growth is demonstrated by the presence of inert PG at the sidewalls and incorporation of fresh PG in the poles during elongation [9]. MreB also localizes at the main growth zones of *Hyphomonas neptunium*, a budding bacterium, co‐localizing with PG turnover (HADA labeling) [67]. In comparison, the apical (tip) growth seen in *F. muscicola* hormogonia or young branches does not resemble zonal growth of one cell; rather, it seems as polar growth of apical cells in a trichome. Apical cell growth was recently also demonstrated in the trichome‐forming subsection III cyanobacteria *Arthrospira* sp. FACHB 792 and *Oscillatoria animalis* and the subsection V cyanobacterium *Mastigocoleus testarum* which forms polar calcicytes [44, 68], demonstrating that this growth mode is present in other cyanobacteria. Our results thus suggest a mixed growth mode for *F. muscicola* where newly growing trichomes (young branches, apical cells after necridia breakage and hormogonia) grow apically by lateral wall expansion while, in mature trichomes, only the septal PG biogenesis takes place. The absence of apical and lateral HADA staining in *F. muscicola* furthermore hints for local differences in PG biogenesis with lipid II exposure at the tips and the lateral cell wall (stained by Van‐FL; [9]) while transpeptidase activity is mainly localized to the septal sites (stained by HADA; [47]). All cell stages of *C. fritschii* displayed only one mode of cell growth, resembling that of coccoid bacteria such as *Synechocystis* [47] and *S. pneumoniae* [9] where PG synthesis occurs solely at the septa. The differences in hormogonia growth modes between the two Stigonematalean species studied here suggest a functional divergence of the cell division machinery within this group. In general, all investigated cyanobacteria so far showed at least a partial septal growth pattern [44, 61, 63]. MreB was found to be dispensable for viability in *Anabaena* [24], suggesting that MreB does not have an essential role in cell wall biogenesis in multicellular cyanobacteria, unlike what is known for many other rod‐shaped bacteria [69]. This is in accordance with the lack of *mreB* in some cyanobacterial strains (Fig. 1B) as well as the apical growth mode in Actinobacteria where MreB is not required for cell wall growth [44]. Notably, we were not able to express *mreB* from *F. muscicola*, *C. fritschii* or *Anabaena* in *Synechocystis*, which lacks MreB, suggesting that MreB has a negative effect on the sphere‐shaped *Synechocystis*. Considering this, one would expect that *mreB* could be deleted in *F. muscicola* and *C. fritschii*, however, proper genome modification systems are yet lacking for those strains [36], which is the likely cause for the failure to produce *F. muscicola* and *C. fritschii mreB* deletion strains. The role of MreB in cell polarity was recently reported in *E. coli*, where the places devoid of MreB become cell poles, allowing the cells to elongate bidirectionally [70]. Using immunofluorescence, we observed diffuse localizations of MreB_Fm_‐His in young branches of *F. muscicola* which coincide with the prominent lateral/apical Van‐FL staining pattern in those types of cells, hinting that MreB might be particularly important for the lateral and apical cell growth of these comparably rapidly growing cell types.

The septal localization of MreB in *Anabaena* and the lateral filaments of GFP‐MreB observed in *F. muscicola*, *C. fritschii*, and *Anabaena* as well as the radiation of GFP‐MreB filaments from the poles in *C. fritschii* suggested that MreB may interact with proteins from the septal junctions. These are cell–cell joining structures of apparently proteinaceous nature connecting two contiguous cells within a trichome in heterocyst‐forming cyanobacteria that aide in the maintenance of the multicellular trichome structure [51, 52, 61, 71, 72]. Septal localization of MreB has been shown during cell division in *C. crescentus*, *E. coli*, and *B. subtilis*, suggesting a role in PG deposition at the cell division septa during cell division [8, 73, 74, 75]. Septal GFP‐MreB_Ana_ spots in *Anabaena* (Fig. 3) were also previously described by [24] and are reminiscent to the septal localization of mYpet–MreB in rod‐shaped bacteria such as *E. coli* [53]. However, we could not identify any interaction of MreB from any of the three cyanobacterial species tested with SepJ, as such, it is unlikely that the presumed cell–cell traversing GFP‐MreB filaments actually traverse through the septal junctions and rather end close to the septal sites. We note that there are other septal junction proteins, such as FraD [72], and other presumed septal junction proteins including FraC [52], and it is possible that MreB could associate with these proteins instead. This is also in accordance with the recently identified width of the septal junctions in *Anabaena*, which was reported to be 7 nm [72], thus precluding the passage of GFP‐MreB, which has an approximate width of 7.4 nm (MreB filaments are reported to be 5 nm in width [76] and GFP is about 2.4 nm in width [77]). The role of MreB in cell division was demonstrated in *E. coli*, where a direct interaction between MreB and FtsZ was shown [53]. However, so far there are no reports of direct MreB‐FtsZ interaction in cyanobacteria. Although in *C. fritschii*, MreB did not localize to the mid‐cell ring, we found that it was able to interact with FtsZ *in vivo* using co‐IP experiments. MreB_Fm_ also occasionally localized to the mid‐cell as rings in young *F. muscicola* branches and MreB_Fm_ and FtsZ_Fm_ were found to directly interact based on BACTH assay results. The fact that we could not identify any interaction of *C. fritschii* FtsZ and MreB in the BACTH assay suggests that the employed T25 and T18 tags in this essay might tamper with proper binding of both proteins, while GFP, which we equipped with a comparably long 12 amino acid linker, might be more flexible allowing for the interaction identified in the co‐IP assays. The interaction of FtsZ with MreB supports the idea of an involvement of MreB with the cell division machinery in subsection V cyanobacteria. However, MreB did not interact with FtsZ in *Anabaena*, supporting the finding that cell division is not impaired in the *mreB* mutant in *Anabaena* [24]. We speculate that an interaction of MreB and FtsZ might be important for the formation of the true branching (*F. muscicola*) and the multiseriate phenotype (*C. fritschii*) in cyanobacteria but not for the division in one plane as seen in *Anabaena*.

Our results show that the different cyanobacterial morphotypes likely vary in their sensitivity toward FtsZ dosage. Such an effect is observed in polar growing bacteria that lack MreB but encode for DivIVA, a tip protein responsible for polar growth in Gram‐positive bacteria [78]. For example, fluctuating FtsZ levels have a severe impact in *C. glutamicum* and *M. tuberculosis*, suggesting that cell division in these organisms is sensitive to the intracellular levels of FtsZ [79, 80, 81]. In *C. glutamicum*, overdose of FtsZ is lethal; up to ×2.6‐fold FtsZ results in viable cells but with aberrant phenotype (cells widen) and a ×4 fold decrease in FtsZ levels lead to filamentous cells (*i.e*.*,* inhibition of cell division) [81]. The formation of buds and branches in species extending by tip growth can be induced by the misplacement of the PG–synthesis complex from the tips during cell enlargement [55, 82, 83]. In our *in vivo* expression experiments, elevated levels of FtsZ readily induced increases in cell sizes and swollen cell morphotypes, which indicates that cell division is impaired as a result of the high FtsZ levels and that cell growth, possibly mediated by MreB [9], proceeds without cell septation, ultimately leading to an increase in cell volume. This phenomenon was previously observed for *Synechocystis* and *Anabaena zipN* knockdown strains [33, 84] as well as for an *ftsZ* knockdown *Anabaena* strain [31] or an *Anabaena* strain predominantly expressing a N‐terminally truncated FtsZ variant [85], indicating that proper divisome function is key for the equilibrium between cell elongation/growth and cell division. Interestingly, the swollen/enlarged phenotypes in *Anabaena* and *F. muscicola* rather resemble the swollen cell phenotypes associated with divisome disturbances in *Synechocy*stis [26, 34] than in *Synechococcus* [27, 56]. Consequently, *Anabaena* and *F. muscicola*, despite their normally rectangular (elliptical) cell shapes, behave more like a sphere than a rod in terms of cell division‐cell elongation balances. Besides, in *F. muscicola* we also observed a higher sensitivity to FtsZ abundance compared to *Anabaena*. Bud‐like multiseriate growth was observed in *F. muscicola* and *Anabaena* upon overexpression of *ftsZ* from P_ftsZ_ and P_glnA_, although, unlike *F. muscicola*, *Anabaena* did not reveal morphological alterations upon overexpression from P_petE_. This, together with the inability of *F. muscicola* cells to grow in liquid culture or to higher density upon overexpressing of *ftsZ‐gfp* from P_ftsZ_ and P_glnA,_ suggests that both promoters are stronger than P_petE_. The lack of liquid culture growth also precluded quantification of FtsZ protein levels in cells expressing *ftsZ* from the different promoters; therefore, this observation can only be supported by the different extends of morphological cell and trichome changes (see Fig. 6A,C). Consequently, *ftsZ* is possibly more tightly regulated either at the transcriptional or posttranscriptional level in subsection V cyanobacteria, highlighting a fundamental difference in the regulation of cell cycle between Subsection IV and V cyanobacteria.

Our *in vitro* FtsZ degradation assays showed a proteolytic activity toward FtsZ in all three tested cyanobacteria, while being notably stronger in *F. muscicola* and *C. fritschii* than in *Anabaena*. *In vitro* proteolysis of FtsZ from a nonidentified protease has previously been reported for *Anabaena* [57]. Here, we showed that the proteolytic activity from *Anabaena* does not act against *F. muscicola* FtsZ but only against *Anabaena* FtsZ and could be inhibited by serine and cysteine protease inhibitors and stimulated by supplementation with Mg^2+^ ions. A novel Ca^2+^‐stimulated serine protease was found to be active in *Westiellopsis ramose* sp. nov. and was also found to be inhibited by protease inhibitors used in our PIC [86]. The precise targets for this protease remain to be identified but it is conceivable that a similar protease could act against FtsZ in *Anabaena*. The proteolytic activity found in *F. muscicola* and *C. fritschii* extracts remained in the presence of PIC and degradation of FtsZ in both strains could only be mitigated upon supplementation with EDTA/EGTA. These results indicate the presence of an additional FtsZ‐specific protease(s) (likely metalloprotease(es)), in members of subsection V cyanobacteria other than serine and cysteine proteases, which seem to be responsible for FtsZ degradation in (Subsection IV) *Anabaena*. We did not identify the nature of *F. muscicola* and *C. fritschii* protease(s); however, the ClpXP (serine) protease has been reported to regulate FtsZ polymerization dynamics in *E. coli*, *B. subtilis*, and *C. crescentus* [20, 87, 88, 89] and direct evidence of degradation of FtsZ monomers was reported in *E. coli* [20, 88]. Given the universal role of ClpXP, which is also encoded in the *F. muscicola* genome, we cannot rule out its function in FtsZ degradation. However, the regulation of FtsZ in cyanobacteria seems to be more complex. In *S. elongatus*, which encodes for three *clpP* genes, deletion of the *clpX* and *clpP1* protease genes leads to filamentation [90]; thus, these proteases may be related to cell division. Notwithstanding, KaiC—the main circadian clock protein—seems to be an important regulator of FtsZ by indirectly affecting FtsZ polymerization [91]. The strong proteolytic activity toward *F. muscicola* and *C. fritschii* FtsZ shows that posttranslational regulation plays an important role in *F. muscicola* and *C. fritschii* development and could indicate that FtsZ proteolysis plays an important role in the branching phenotype. Furthermore, *Anabaena* cells strongly overexpressing *ftsZ* formed branch‐like formations. Consequently, we propose that FtsZ is tightly regulated at trichomes undergoing binary fission, so that a higher dose of FtsZ leads to multiple ring formation and branching or multiseriate trichome growth. A higher protease activity would keep FtsZ levels low to avoid multiple divisions whereas a higher FtsZ dosage will overrun the available protease and lead to multiple ring formation blocking cell division. Attempts to generate *ftsZ* deletion or knockdown strains remained unsuccessful, which could serve as a further indication for strictly regulated FtsZ levels and which would be in concert with the essentiality of FtsZ for cyanobacterial viability [26, 27, 29, 30].

## Materials and methods

### Sequence analysis

Protein families of cyanobacteria were extracted from [92] (see File S1 for accession numbers). Protein sequences were aligned with mafft [93] and the % identical amino acids were calculated as the average pairwise sequence similarity using an in‐house matlab ^©^ script (created by Tal Dagan, University of Kiel, Germany). The threshold for sequence similarity (and as such homology) was set to ≥ 30%.

### Bacterial strains and growth conditions

*Fischerella muscicola* PCC 7414, *Chlorogloeopsis fritschii* PCC 6912, and *Anabaena* sp. PCC 7120 were obtained from the Pasteur Culture Collection (PCC) of cyanobacteria, France. Stock cultures were grown photoautotrophically at a 18‐h/6‐h light/dark regime in liquid BG11 or BG11_0_ medium at 37 °C (*F. muscicola* and *C. fritschii*) or 30 °C (*Anabaena*) with a light intensity of 30 µmol·m^−2^·s^−1^. *Escherichia coli* strains XL1‐blue, BL21 (DE3), HB101, and ED8654 were used for cloning and conjugation. *E. coli* BTH101 was used for BACTH assays and *E. coli* cells were grown at 37 °C in standard LB growth medium supplemented, if appropriate, with 100 µg·mL^−1^ ampicillin (Amp), 50 µg·mL^−1^ kanamycin (Km), or 25 µg·mL^−1^ chloramphenicol (Cm). Antibiotic selection for cyanobacteria was 30 µg·mL^−1^ neomycin (Nm).

### DNA isolation, amplification, cloning, and conjugation

DNA isolations from *F. muscicola* and *C. fritschii* were performed from 50 mL cell culture (pelleted and frozen in liquid nitrogen) by grinding with mortar and pestle following a protocol for plants with high polysaccharide content [94]. All PCRs were performed with Phusion or Q5 polymerase (New England Biolabs, Frankfurt am Main, Germany) with primers described in Table S1. Cloning was either done using Gibson assembly or restriction enzyme‐based procedures. Sequence integrity of generated plasmids was verified by Sanger sequencing (Eurofins, Ebersberg, Germany). Genes and promoter regions were amplified from genomic DNA; *gfpmut3.1* was amplified from pRL153‐GFP [95]. A silent nucleotide exchange (CAT to CAC) was introduced in the coding sequence of *gfpmut3.1* to remove a *NdeI* restriction site. Detailed plasmid construction procedures can be requested from the authors. Plasmids and strains used or generated in this study are listed in Table S1. All plasmids were transferred into cyanobacteria by conjugation as previously described [36, 96].

### *ftsZ* overexpression and purification

Polyhistidine tagged *ftsZ* from *F. muscicola*, *C. fritschii*, and *Anabaena* was expressed in *E. coli* BL21 (DE3) cells with 0.5 mm IPTG for 3 h at 37 °C. *E. coli* cells were lysed using a Precellys 24 homogenizer with 0.1‐mm glass beads in native lysis buffer (50 mm NaH_2_PO_4_, 300 mm NaCl, 1% Triton X‐100, pH 8.0) and His‐tagged FtsZ was purified by affinity chromatography using Ni‐NTA resins (Thermo Fisher Scientific, Dreieich, Germany). Subsequently, FtsZ‐His protein concentration was measured using Bradford assay (Bio‐Rad, Feldkirchen, Germany) and FtsZ was either directly used for *in vitro* FtsZ polymerization assays as described below or dialyzed into FtsZ storage buffer (50 mm Tris pH 7.5, 1 mm EDTA, 250 mm NaCl, 10% glycerol) and stored at −80 °C until further use.

### *In vitro* FtsZ polymerization assay

One hundred microlitre of 1 mg·mL^−1^ of Ni‐NTA‐purified FtsZ‐His was applied to Zeba Spin Columns (7K MWCO) and transferred into HLB (Hepes labeling buffer; 25 mm Hepes, 150 mm NaCl, pH 7.4). Afterward, 1 µL of 1 mg NHS‐Fluorescein in 100 µL DMSO was added and incubated at RT for 1 h in the dark. Labeled proteins were then transferred to FtsZ polymerization buffer (FPB; 50 mm MES, 50 mm KCl 10 mm MgCl_2_, 2 mm GTP, pH 6.5) using Zeba Spin Columns (7K MWCO) and incubated for 40 min at 30 °C before analysis of *in vitro* FtsZ filaments by epifluorescence microscopy.

### Antibody synthesis

Polyclonal rabbit antibodies, raised against the peptides TLDNNQGLTYKNSQS and LDIPDFLRKRTPPRN corresponding to the N and C terminus of *F. muscicola* and *C. fritschii* FtsZ, were obtained by Eurogentec (Seraing, Belgium).

### Total protein extraction and immunoblotting

Protein extracts were obtained from 30 to 40 mL of cyanobacterial cultures pelleted by centrifugation for 10 min at 4800 ***g*** at RT. Cell pellets were resuspended in 1 mL of the respective lysis buffer (CLB: 50 mm HEPES, 150 mm NaCl, 5% glycerin, 1% Triton X‐100; CLB supplemented with 10 mm MgCl_2_) supplemented with 1 mm DTT and were indicated with protease inhibitor cocktail (1× cOmplete, EDTA‐free; Roche, Mannheim, Germany) and 5 mm EDTA and 5 mm EGTA. Cells were lysed by homogenization with a Precellys 24 homogenizer (Bertin Instruments, Frankfurt am Main, Germany) in VK05 (*Anabaena*) or SK38 (*F. muscicola* and *C. fritschii*) lysis tubes with 3 × 30 s at 6500 r.p.m. Cell debris was removed by centrifugation for 10 min at 21 000 ***g*** and 4 °C, and protein concentration was determined with Bradford reagent (Bio‐Rad). Proteins were separated by SDS/polyacrylamide gel electrophoresis and then transferred onto nitrocellulose membranes. Detection of FtsZ was then done using polyclonal rabbit anti‐FtsZ_SubsV_ (1 : 2000 dilution) or polyclonal rabbit anti‐RbcL (Agrisera, Vännäs, Sweden; large subunit forms I and II; 1 : 10 000 dilution) primary antibody.

### Co‐immunoprecipitation

For co‐immunoprecipitations, *F. muscicola*, *C. fritschii*, and *Anabaena* WT or strains expressing *gfp‐mreB* from P_mreB_ were grown in BG11 liquid medium. About 40 mL cell suspensions were harvested by centrifugation (4800 ***g***, RT, 10 min) and washed once in 40 mL PBS (137 mm NaCl, 2.7 mm KCl, 10 mm phosphate buffer). Anti‐GFP co‐immunoprecipitation of cell‐free lysates was then performed using the μMACS GFP isolation kit (Miltenyi Biotec, Bergisch Gladbach, Germany). For this, cells were pelleted by centrifugation and resuspended in 1 mL PBS‐N (PBS supplemented with 1% NP‐40) lysis buffers supplemented with a protease inhibitor cocktail (cOmplete™, EDTA‐free Protease Inhibitor Cocktail, Sigma‐Aldrich, Mannheim, Germany). Cells were lysed by bead‐beating using a Precellys 24 homogenizer (3 × 30 s, 6500 r.p.m.) (Bertin Instruments, Frankfurt am Main, Germany) and the VK05 (*Anabaena*) or SK38 (*F. muscicola* and *C. fritschii*) lysis kits (Bertin). The lysates were centrifuged (21 000 ***g***, 4 °C, 10 min) and cell‐free supernatants were incubated with µMACS anti‐GFP microbeads (Miltenyi Biotec, Bergisch Gladbach, Germany) for 30 min on ice. Afterward, the solutions were applied to µColumns (Miltenyi Biotec) and then washed 3× with 1 mL PBS‐N. Finally, proteins were eluted in elution buffer [50 mm Tris/HCl (pH 6.8), 50 mm DTT, 1% SDS, 1 mm EDTA, 0.005% bromphenol blue, 10% glycerol].

### FtsZ degradation assays

*In vitro* degradation of native FtsZ was analyzed by incubating cell‐free extracts for 3 h at 37 °C (*F. muscicola* and *C. fritschii*) or 30 °C (*Anabaena*). Samples containing 75 µg fresh total proteins were taken at indicated intervals for western blot analysis with rabbit anti‐FtsZ_SubsV_ antibody as described above. Reactions were stopped by adding Laemmli buffer supplemented with 25 mm EDTA and incubating for 10 min at 95 °C.

*In vitro* degradation of purified FtsZ_Fm_‐His was assayed by incubating 1 µg FtsZ_Fm_‐His per 75 µg total cell‐free extracts from *F. muscicola*, *C. fritschii*, and *Anabaena* with (or without) protease inhibitors and with (or without 5 mm EDTA and EGTA) at 37 °C (*F. muscicola* and *C. fritschii*) or 30 °C (*Anabaena*) for 3 h, and samples were taken at indicated intervals. Reactions were stopped by adding Laemmli buffer supplemented with 25 mm EDTA and incubation at 95 °C for 10 min. FtsZ_Fm_‐His degradation was analyzed by western blot analysis using monoclonal mouse anti‐His (Thermo Fischer Scientific; 1 : 5000 dilution) primary antibody.

### Immunofluorescence

For immunofluorescence of *Anabaena* FtsZ, 100–200 µL of cells was dried on poly‐l‐lysine‐coated microscope slides (Electron Microscopy Sciences, Munich, Germany) at 37 °C. For fixation, slides were placed on Petri dishes, submerged in 70% ice‐cold ethanol, and incubated for 30 min at −20 °C. After rinsing with PBS (137 mm NaCl, 2.7 mm KCl, 10 mm phosphate buffer), samples were blocked with 3% BSA in PBS‐T [PBS with 0.1% (v/v) Tween 20] for 45 min at room temperature (RT). Cells were then incubated with rabbit anti‐FtsZ_SubsV_ (1 : 100 dilution) primary antibody in 3% BSA in PBS‐T for 90 min at RT. The samples were supplemented with 7.5 µg·mL^−1^ Alexa‐488‐conjugated anti‐rabbit secondary antibody (Invitrogen, Dreieich, Germany) and incubated for 45 min at 30 °C in darkness. The incubation was followed by three washing steps for 5 min in PBS‐T buffer under mild agitation. Afterward, samples were covered with a drop of Prolong Diamond (Thermo Fischer Scientific, Dreieich, Germany) and analyzed by epifluorescence microscopy (Zeiss Axio Imager 2; Plan‐Apochromat 63×/1.40 Oil DIC M27 objective, Oberkochen, Germany).

For immunofluorescence of *F. muscicola* and *C. fritschii* FtsZ, a modified protocol to the above‐described protocol was employed to overcome obstacles associated with their comparably resistant cell envelope. All procedures were performed in 1.5‐mL reaction tubes. Initially, about 1 mL cell suspensions were harvested by centrifugation (6500 ***g***, 4 min, RT), washed once with PBS‐T, and then incubated for 30 min at −20 °C with 70% ice‐cold ethanol. Cells were washed two times with PBS‐T and incubated for 30 min at RT with mild agitation in PBS supplemented with 0.05% Triton X‐100. Cells were pelleted, the supernatant was removed, and the cell pellet was resuspended in lysozyme buffer (50 mm Tris/HCl pH 7.4, 50 mm NaCl, 5 mm EDTA, and 2 mg·mL^−1^ lysozyme) and incubated for 30 min at 37 °C with mild agitation. Afterward, cells were washed 3× in PBS‐T and unspecific binding sites were then blocked for 45 min at RT in PBS‐T supplemented with 1% BSA with mild agitation. Cells were then incubated with rabbit anti‐FtsZ_SubsV_ (1 : 100 dilution) or mouse anti‐His (1 : 200 dilution; Thermo Fischer Scientific) primary antibody in 1% BSA in PBS‐T for 90 min at 30 °C. This was followed by four washing steps with PBS‐T and incubation with 7.5 µg·mL^−1^ Alexa‐488‐conjugated anti‐rabbit or anti‐mouse secondary antibody and incubated for 1 h at 30 °C in darkness. Finally, cells were washed 4× with PBS‐T and analyzed by epifluorescence microscopy.

### Fluorescence labeling of active sites of cell wall synthesis

Fluorescently labeled vancomycin (BODIPY^®^ FL Vancomycin; Thermo Fisher Scientific) was employed to visualize sites of active PG synthesis in exponentially growing cyanobacterial cultures. 5 µg·mL^−1^ BODIPY^®^ FL Vancomycin (Van‐FL) was added to 200 µL cells from liquid culture and incubated in darkness for 1 h at RT similar to what was described earlier [61]. To remove unbound Van‐FL, cells were washed three times with 1 mL BG11 by centrifugation (4700 ***g***, 7 min, RT). Van‐FL localization was visualized by epifluorescence microscopy. For labeling with HADA, a 100 mm stock solution of HADA (kindly provided by Michael S. VanNieuwenhze, Indiana University, USA) in DMSO was prepared and 200 µm HADA (final concentration) was added to *F. muscicola*, *C. fritschii*, or *Anabaena* grown in BG11 or to cells 3× washed in 1 mL BG11_0_ and then resuspended in BG11_0_. Cells were grown at standard growth conditions for 4 days before visualization.

### Microscopy

For epifluorescence microscopy, an Axio Imager.M2 light microscope (Carl Zeiss, Oberkochen, Germany) equipped with Plan‐Apochromat 63×/1.40 Oil M27 objective and the AxioCam MR R3 imaging device (Carl Zeiss) was used. GFP, Alexa Fluor 488, and BODIPY™ FL Vancomycin (Van‐FL) fluorescence was visualized using filter set 38 [Carl Zeiss; excitation: 470/40 nm band pass (BP) filter; emission: 525/50 nm BP]. HADA fluorescence was visualized using filter set 49 (Carl Zeiss; G365 excitation filter; emission 445/50 nm BP). Chlorophyll autofluorescence was recorded using filter set 15 (Carl Zeiss; excitation: 546/12 nm BP; emission: 590 nm long pass).

For visualization of *E. coli* expressing FtsZ‐GFP or GFP‐MreB from the respective native promoters, *E. coli* were grown overnight at 37 °C and observed by epifluorescence microscopy the next day. For visualization of *E. coli* cells expressing FtsZ‐His, cells were grown overnight at 37 °C, diluted 1 : 40 in fresh LB medium, grown for 2 h at 37 °C, and then induced with 0.5 mm IPTG for 3 h at 37 °C. FtsZ‐induced filamentation of *E. coli* cells was then assessed by bright‐field microscopy.

## Conflict of interest

The authors declare no conflict of interest.

## Author contributions

BLS, KS, and JW performed the genetic and cell biology analyses and conducted the microscopy studies. RK performed comparative sequence analyses and FS designed graphical illustrations. KS and BLS designed the research; BLS and KS wrote the manuscript.

## Supporting information


**Fig. S1.** Van‐FL controls. Merged chlorophyll autofluorescence and Van‐FL fluorescence and bright‐field micrographs of *F. muscicola*, *C. fritschii*, and *Anabaena* cells grown in the presence of 5 µg ml^‐1^ unlabeled vancomycin to indicate background fluorescence when cells are excited with the filter set 38 (Carl Zeiss). Scale bars: 10 µm.

**Fig. S2.** Staining of cyanobacterial strains with HADA. (A‐H) Bright‐field and merged chlorophyll autofluorescence (red) and HADA fluorescence micrographs of (A‐D) *F. muscicola*, (E‐G) *C. fritschii* and (H) *Anabaena* cells stained with HADA. Micrographs indicate different growth stages of the respective cyanobacterium: (A) *F. muscicola* hormogonia, (B,C,D) *F. muscicola* mature trichomes with (B) branches, (C) heterocysts or (D) necridia (i.e., dead cells that mark sites of hormogonia release, indicated by an orange triangle), (E‐G) *C. fritschii* mature multiseriate trichomes with (F) hormogonia or (G) heterocysts and (H) *Anabaena* mature trichome with heterocysts. White arrows indicate heterocysts. Scale bars: 10 µm.

**Fig. S3.** GFP fluorescence controls. Merged chlorophyll autofluorescence (red) and GFP fluorescence and bright‐field micrographs of *F. muscicola*, *C. fritschii* and *Anabaena* cells expressing *gfp* from P_petE_. Scale bars: 10 µm.

**Fig. S4.** Immunolocalization of MreB‐His in *F. muscicola*. Alexa‐488 and bright‐field micrographs of anti‐His immunofluorescence staining of (A‐C) *F. muscicola* expressing polyhistidine tagged *mreB* from P_petE_ and of (D) *F. muscicola* WT. White triangles mark mid‐cell localization of MreB‐His. Scale bars: 10 mm.

**Fig. S5.** Properties of cyanobacterial FtsZ and MreB. (A,C) Merged GFP fluorescence and bright‐field micrographs of *E. coli* cells expressing *ftsZ* or *mreB* from *F. muscicola*, *C. fritschii* or *Anabaena*: (A) *gfp‐mreB* or (C) *ftsZ‐gfp* from (A) P_mreB_ or (C) P_ftsZ_ of. (B) Bright‐field micrographs of *E. coli* cells expressing polyhistidine tagged *ftsZ*_Fm_, *ftsZ*_Cf_ or *ftsZ*_Ana_ from the P_T7_ promoter. (D) NHS‐Fluorescein fluorescence micrographs of purified FtsZ_Fm_‐His, FtsZ_Cf_‐His or FtsZ_Ana_‐His labeled with an excess of NHS‐Fluorescein. Scale bars: (A,C) 5 µm or (B,D) 10 µm.

**Fig. S6.** MreB does not interact with SepJ. Beta‐galactosidase assays of *E. coli* cells co‐expressing indicated translational fusion constructs of all possible pairwise combinations of *mreB* with *sepJ*. Quantity values are given in Miller Units per milligram LacZ of the mean results from three independent colonies. Error bars indicate standard deviations (n = 3). Neg: pKNT25 plasmid carrying *mreB* co‐transformed with empty pUT18C. Pos: Zip/Zip control. Values indicated with ns are not significantly different from the negative control. (Dunnett's multiple comparison test and one‐way ANOVA).

**Fig. S7.** Multiseriate growth and minicell formation upon excess of FtsZ in *Anabaena*. Merged chlorophyll autofluorescence (red) and GFP fluorescence and bright‐field micrographs of *Anabaena* expressing GFP‐FtsZ^I280F^ or *gfp‐ftsZ* from P_glnA_. Note: despite lack of proper FtsZ polymerization into Z‐rings in the GFP‐FtsZ^I280F^ variant, multiseriate growth and minicells can still be detected. Scale bars: 10 µm.

**Table S1.** Oligos, strains and plasmids.

**File S1.** Presence/absence of FtsZ, FtsQ, and MreBCD homologs in cyanobacteria.

**File S2.** Quantification of MreB localization patterns.

**Video S1.** Localization of GFP‐MreB_Fm_ filaments in *E. coli*.

**Video S2.** Localization of GFP‐MreB_Cf_ filaments in *E. coli*.

**Video S3.** Localization of GFP‐MreB_Ana_ filaments in *E. coli*.

## Data Availability

The data that support the findings of this study are available from the corresponding authors upon reasonable request.
