# Supplementary figures and images for: The role of the cytoskeletal proteins MreB and FtsZ in multicellular cyanobacteria
Source: FEBS Open Bio. 2020 Nov 13;10(12):2510–31. doi: 10.1002/2211-5463.13016 (PMC7714070; doi:10.1002/2211-5463.13016)

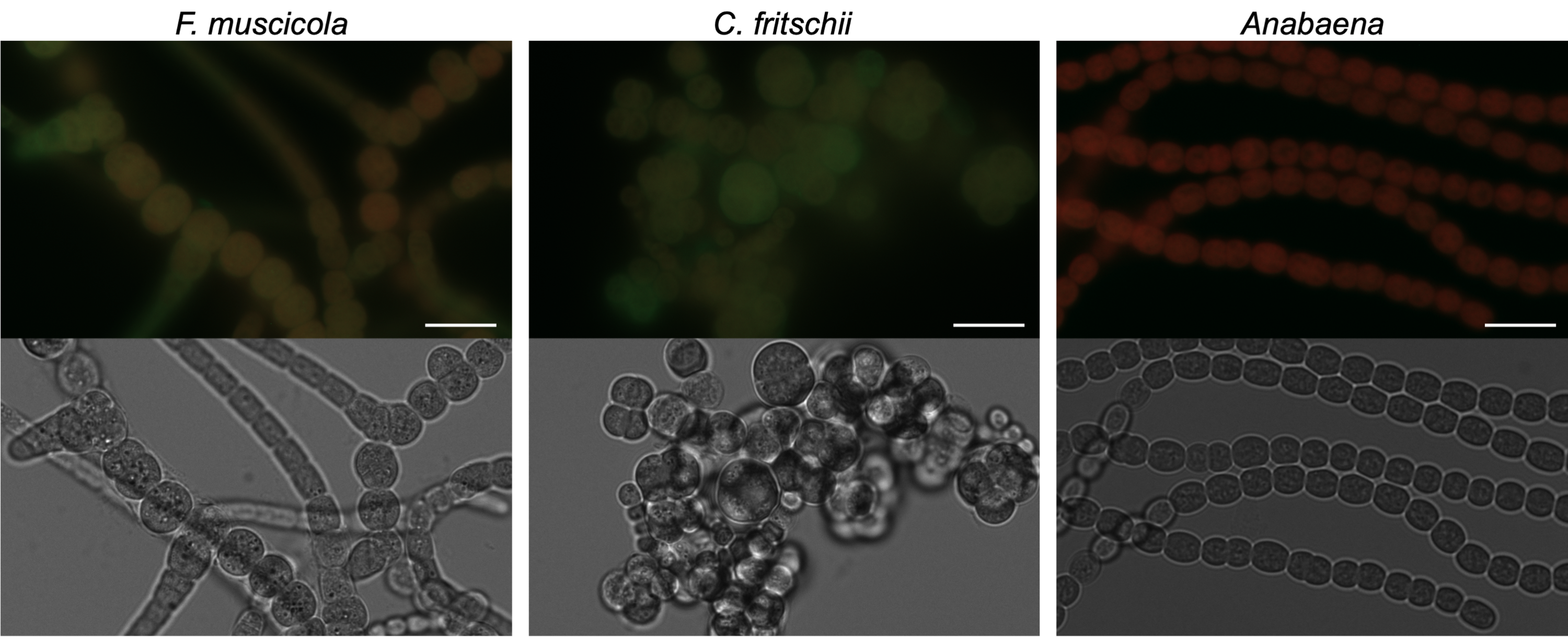

Supplement: Supplementary file 1 — Fig. S1. Van‐FL controls. Merged chlorophyll autofluorescence and Van‐FL fluorescence and bright‐field micrographs of F. muscicola, C. fritschii, and Anabaena cells grown in the presence of 5 µg ml‐1 unlabeled vancomycin to indicate background fluorescence when cells are excited with the filter set 38 (Carl Zeiss). Scale bars: 10 µm. [file FEB4-10-2510-s001.tiff]

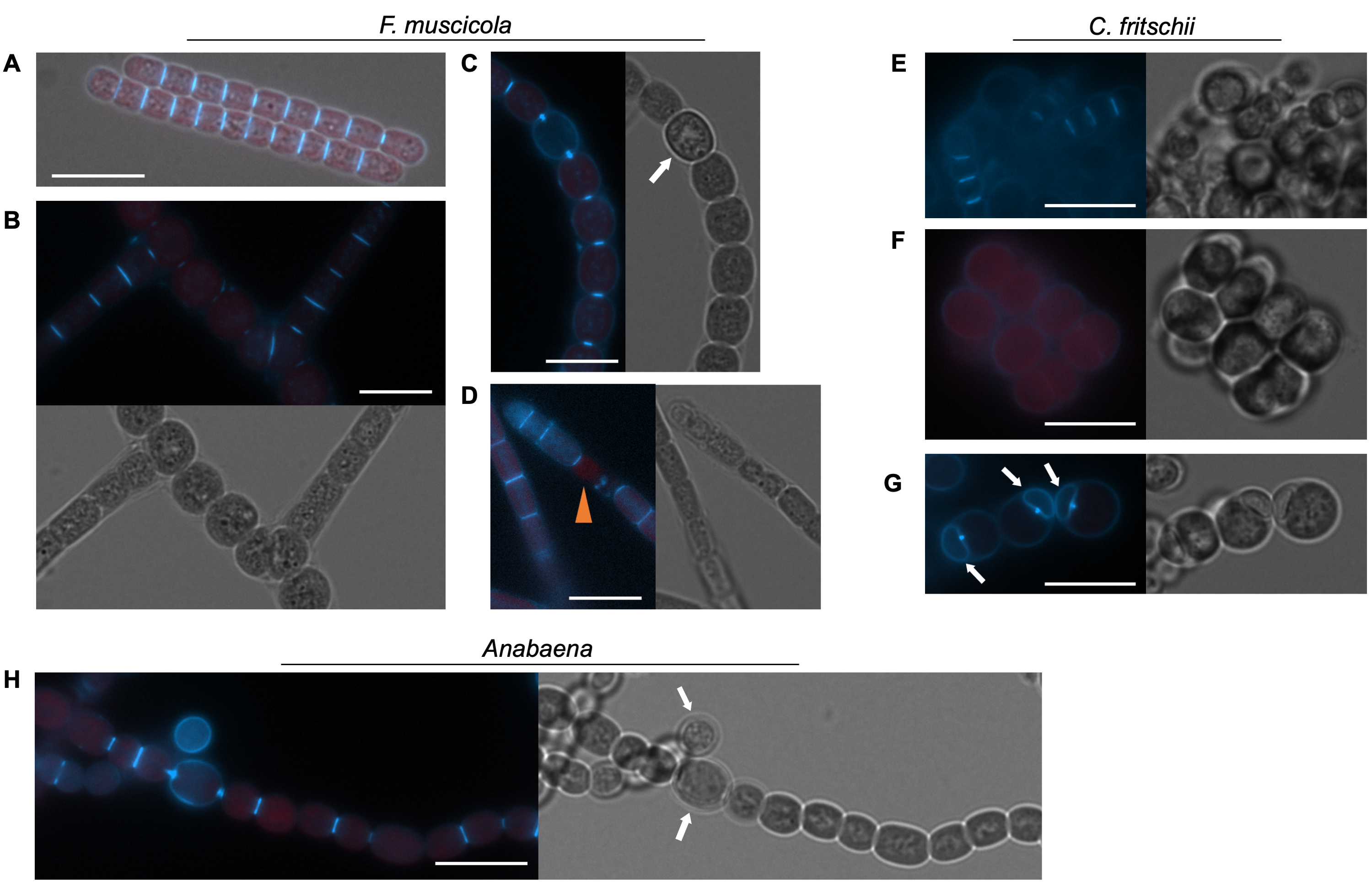

Supplement: Supplementary file 2 — Fig. S2. Staining of cyanobacterial strains with HADA. (A‐H) Bright‐field and merged chlorophyll autofluorescence (red) and HADA fluorescence micrographs of (A‐D) F. muscicola, (E‐G) C. fritschii and (H) Anabaena cells stained with HADA. Micrographs indicate different growth stages of the respective cyanobacterium: (A) F. muscicola hormogonia, (B,C,D) F. muscicola mature trichomes with (B) branches, (C) heterocysts or (D) necridia (i.e., dead cells that mark sites of hormogonia release, indicated by an orange triangle), (E‐G) C. fritschii mature multiseriate trichomes with (F) hormogonia or (G) heterocysts and (H) Anabaena mature trichome with heterocysts. White arrows indicate heterocysts. Scale bars: 10 µm. [file FEB4-10-2510-s002.tiff]

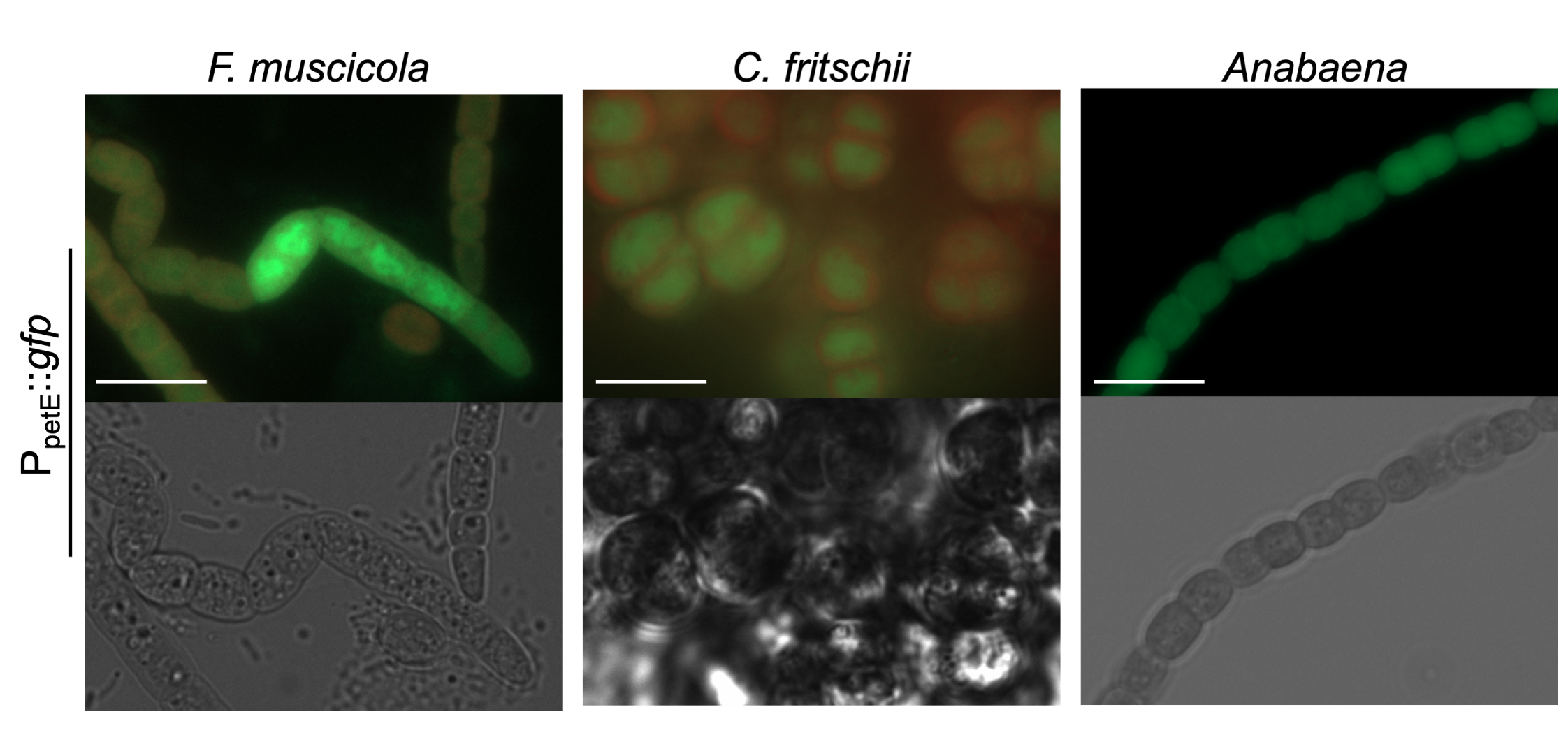

Supplement: Supplementary file 3 — Fig. S3. GFP fluorescence controls. Merged chlorophyll autofluorescence (red) and GFP fluorescence and bright‐field micrographs of F. muscicola, C. fritschii and Anabaena cells expressing gfp from PpetE. Scale bars: 10 µm. [file FEB4-10-2510-s003.tiff]

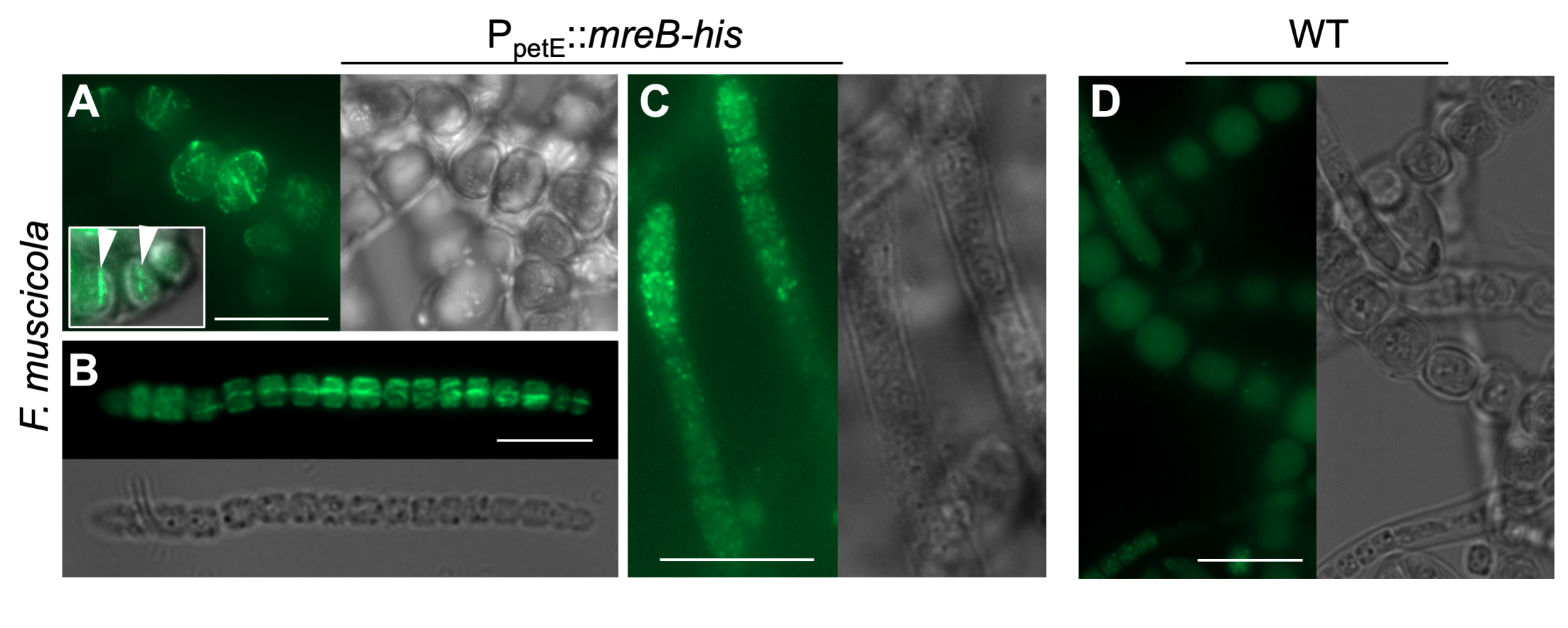

Supplement: Supplementary file 4 — Fig. S4. Immunolocalization of MreB‐His in F. muscicola. Alexa‐488 and bright‐field micrographs of anti‐His immunofluorescence staining of (A‐C) F. muscicola expressing polyhistidine tagged mreB from PpetE and of (D) F. muscicola WT. White triangles mark mid‐cell localization of MreB‐His. Scale bars: 10 mm. [file FEB4-10-2510-s004.tiff]

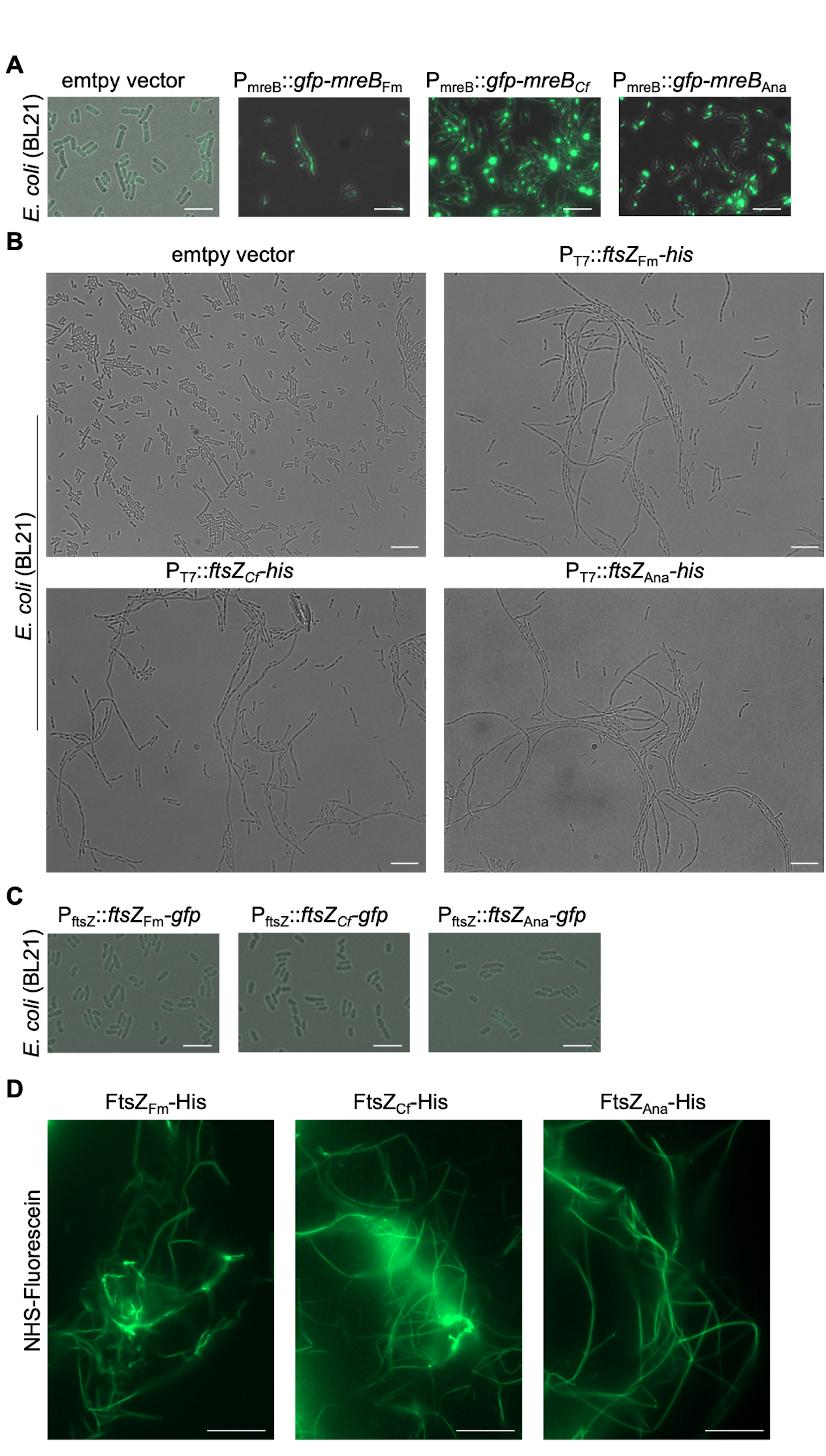

Supplement: Supplementary file 5 — Fig. S5. Properties of cyanobacterial FtsZ and MreB. (A,C) Merged GFP fluorescence and bright‐field micrographs of E. coli cells expressing ftsZ or mreB from F. muscicola, C. fritschii or Anabaena: (A) gfp‐mreB or (C) ftsZ‐gfp from (A) PmreB or (C) PftsZ of. (B) Bright‐field micrographs of E. coli cells expressing polyhistidine tagged ftsZ Fm, ftsZ Cf or ftsZ Ana from the PT7 promoter. (D) NHS‐Fluorescein fluorescence micrographs of purified FtsZFm‐His, FtsZCf‐His or FtsZAna‐His labeled with an excess of NHS‐Fluorescein. Scale bars: (A,C) 5 µm or (B,D) 10 µm. [file FEB4-10-2510-s005.tiff]
