# Supplementary material for: The role of the cytoskeletal proteins MreB and FtsZ in multicellular cyanobacteria
Source: FEBS Open Bio. 2020 Nov 13;10(12):2510–31. doi: 10.1002/2211-5463.13016 (PMC7714070; doi:10.1002/2211-5463.13016)
Supplement: Supplementary file 8 — Table S1. Oligos, strains and plasmids. [file FEB4-10-2510-s008.docx]

**Table S1**. Oligos, strains and plasmids

| **Oligonucleotides** | **Sequence 5’-3’** |
| --- | --- |
| P*petE*_Ana_F_XhoI | GTAGCTCGAGTAGCCTTAAACAAGTAAAAG |
| P*petE*_Ana_R_*gfp* | TCCTTTACGCATACCTGTAGTTTTATTTTTCTTATTTC |
| P*petE*_Ana_F_BamHI | GCAGTAGGATCCTAGCCTTAAACAAGTAAAAG |
| P*petE*_Ana_R_NdeI-SacI | GCAGTAGAGCTCCATATGACCTGTAGTTTTATTTTTCTTATTTC |
| *gfp*F_12AS_SacI | AACTGAGCTCGCTAGTGCATCTGCTAGTGCTAGTGCATCTGCTAGTCGTAAAGGAGAAGAACTT |
| *gfp*F_P*petE*_Ana_ | AAAACTACAGGTATGCGTAAAGGAGAAGAAC |
| *gfp*R_EcoRI | GAGAATTCTTATTTGTATAGTTCATCCAT |
| *gfp*R_12AS_SacI | GCAGTAGAGCTCACTAGCAGATGCACTAGCACTAGCAGATGCACTAGCTTTGTATAGTTCATCCATGCCATG |
| *gfp*R_MCS | CAAGAATTCGAGCTCCCCGGGGGTACCCTTGTATAGTTCATCCATGC |
| *mreB*F_subsV_NdeI | GCTACACATATGGGCCTTTTCAGTAAGTTTTCC |
| *mreB*_Fm_R_SacI_N | GTTGTAGAGCTCAGAATATTTCGGGAACGCCCG |
| *ftsZ*_Fm_F_NheI | AACTGCTAGCACTCTTGATAATAACCAAGGA |
| *ftsZ*_Fm_R_HA-EcoRI | GAATTCTTAAGCGTAGTCTGGGACGTCGTATGGGTAATTACGTGGTGGTGTCCG |
| P*glnA*_Cf_F_BamHI | CATGGATCCTCCTTTTTCTCCCAATCGTTCCT |
| P*glnA*_Cf_R_NdeI | CATATGGGTTACTCCTTCTCTACCGTTTTAG |
| P*ftsZ*_Fm_F_BamHI | ACAGGATCCGATTAAAGCAATCCTAGCCAA |
| P*ftsZ*_Ana_F_BamHI | GGATCCTCATGTGTCAAAAATTCCTTATA |
| P*ftsZ*_Fm_R_NdeI | TACACATATGTGGATTCACAAAGGTAGATAAACGAC |
| P*ftsZ*_Ana_R_NdeI | TACACATATGGATTCGGGAAGGTAGATAAAC |
| *ftsZ*_Fm_F_NdeI | TATACATATGACTCTTGATAATAACCAAGGA |
| *ftsZ*_Fm_R_SacI | GCTAGAGCTCATTACGTGGTGGTGTCCG |
| *ftsZ*_Ana_F_NdeI | CATATGACACTTGATAATAACCAAG |
| *ftsZ*_Ana_R_SacI | GAGCTCATTTTTGGGTGGTCGCC |
| *ftsZ*_Ana_F_SacI | GAGCTCACACTTGATAATAACCAAG |
| *ftsZ*_Ana_R_EcoRI-ClaI | ATCGATGAATTCTTAATTTTTGGGTGGTCGC |
| *ftsZ*_SV_ F_SacI | GAGCTCACWCTYGATAATAACCAAGG |
| *ftsZ*_Fm_R_EcoRI-ClaI | ATCGATGAATTCTTAATTACGTGGTGGTGT |
| C_mreBR_Xba_Eco | GCAGTATCTAGAGAATTCTACATATTGCGGGAACGA |
| F_mreBR_Xba_Eco | GCAGTATCTAGAGAATTCTAAATATTTCGGGAACGC |
| mreBF_subsV_NdeI | GCTACACATATGGGCCTTTTCAGTAAGTTTTCC |
| 6912_mreBR_SacI | GTTGTAGAGCTCCATATTGCGGGAACGACCA |
| 7414_mreBR_SacI | GTTGTAGAGCTCAATATTTCGGGAACGCCCG |
| SmaI-mreBCD_For | GAGAGCCCGGGCTTTTTAGGAACTTTC |
| SacI_mreBCD_Rev | CGTAGAGCTCTAACTACTAACTTTCCAAC |
| 7414_mreBR_BamHI | GGATCCTAAATATTTCGGGAACGC |
| 7120_mreBR_BamHI | GGATCCTACATATTTCGAGATCGTC |
| 6912_mreBR_BamHI | GGATCCTACATATTGCGGGAACGA |
| pmreB7120_F | TTTTGGTCATGAGATTATCAAAAAGTCTAGTTGATCTACTCTAATTACTGGCG |
| pmreB7120_R | AGTTCTTCTCCTTTACGCATGCGTCTCTATGCCCCCTAT |
| GFP_F | ATGCGTAAAGGAGAAGAACTTTTCAC |
| MreB7120_R | GAGGCCCTTTCGTCTTCAAGCTACATATTTCGAGATCGTCCGC |
| pmreB7414_F | TTTTGGTCATGAGATTATCAAAAAGGACTGGGGAAGAACGCAG |
| pmreB7414_R | AGTTCTTCTCCTTTACGCATGGGTTTTGTCGCCCCCTA |
| MreB7414_R | GAGGCCCTTTCGTCTTCAAGCTAAATATTTCGGGAACGCCCG |
| FtsZ6912_pET_A | TGTTTAACTTTAAGAAGGAGATATACATATGACACTCGATAATAACCAAGGG |
| FtsZ6912_pET_B | CAGTGGTGGTGGTGGTGGTGCTCGAGGTTGCGTGGTGGTCGC |
| pftsZ7120_25C_F | TTTTGGTCATGAGATTATCAAAAAGGCACAATTACGCCACATCAAT |
| pftsZ7120_FtsZ_R | CTTGGTTATTATCAAGTGTCATTGGATTCGGGAAGGTAG |
| FtsZ7120_pftsZ_F | CTTCCCGAATCCAATGACACTTGATAATAACCAAGAGC |
| GFP_25C_R | AGGCCCTTTCGTCTTCAAGTTATTTGTATAGTTCATCCATGCCATGTGT |
| pftsZ7414_25C_F | TTTTGGTCATGAGATTATCAAAAAGGATTAAAGCAATCCTAGCCAATCAAGA |
| pftsZ7414_FtsZ_R | CCTTGGTTATTATCAAGAGTCATTGGATTCACAAAGGTAGATAAACG |
| FtsZ7414_pftsZ_F | ATCTACCTTTGTGAATCCAATGACTCTTGATAATAACCAAGGAC |
| pftsZ6912_25C_F | TTTTGGTCATGAGATTATCAAAAAGGGTGTTCCCACCTCCCGATT |
| pftsZ6912_FtsZ_R | GGTTATTATCGAGTGTCATTGGATTCACAAAGGTAGATAAACGAC |
| FtsZ6912_pftsZ_F | TCTACCTTTGTGAATCCAATGACACTCGATAATAACCAAGGG |

Restriction sites are underlined

| **Strain** | **Genotype/Description** | **Reference** |
| --- | --- | --- |
| *F. muscicola* PCC 7414 | WT | PCC strain |
| *C. fritschii* PCC 6912 | WT | PCC strain |
| *Anabaena* sp. PCC 7120 | WT | PCC strain |
|  |  |  |
| **Plasmids** | **Description** | **Reference** |
| pJET1.2/blunt | Cloning vector for *E. coli*. Amp^R^ | Thermo Scientific |
| pBSK(+) | Cloning vector (phagemid) for *E. coli*. Amp^R^ | Stratagene |
| pet21a(+) | Bacterial vector for expressing N- terminal T7 and/or C-terminal His6-tagged proteins in *E. coli* | Novagen |
| pCR^TM^4Blunt-TOPO® | Cloning vector for *E. coli*. Amp^R^, Km^R^ | Invitrogen |
| pRL271 | Suicide vector used for homologous recombination in cyanobacteria; contains *sacB* for positive selection of double recombination events. Cm^R^, Em^R^ | (Black, Cai and Wolk, 1993) |
| pRL25C | Shuttle cosmid vector for cyanobacteria and *E. coli*. Km^R^, Nm^R^ | (Wolk *et al.*, 1988) |
| pRL153-GFP | Shuttle vector for *E. coli* and cyanobacteria carrying the P_trc_::*gfpmut3.1* cloned into the unique NheI site from pRL153 (RSF1010 derivative). Kan^R^ | (Tolonen, Liszt and Hess, 2006) |
| pEGFPC | pJET1.2 based plasmid carrying P_petE_::12AS-*gfpmut3.1** for generation of C-terminal *gfp* fusions. Amp^R^. Flanked by BamHI and EcoRI sites for subcloning in pRL25C | This study |
| pEGFPN | pJET1.2 based plasmid carrying P_petE_::*gfpmut3.1**-12AS for generation of N-terminal *gfp* fusions. Amp^R^. Flanked by BamHI and EcoRI sites for subcloning in pRL25C | This study |
| pBEGFPN | pBSK(+) based plasmid carrying P_petE_::*gfpmut3.1** for generation of N-terminal gfp fusions. Amp^R^. Flanked by XhoI and SacI sites for subcloning in pRL271 | This study |
| pGPTN | pRL25C with P_glnA_::*gfp* inserted between the BamHI-EcoRI sites. Km^R^, Nm^R^. | (Stucken *et al.*, 2012) |
| petMreBFm | pet21a(+) with *mreB*_Fm_ inserted in frame between the NdeI-SacI sites. Amp^R^ | This study |
| petFtsZFm-His | pet21a(+) with *ftsZ*_Fm_ inserted in frame between the NdeI-SacI sites. Amp^R^ | This study |
| petFtsZAna-His | pet21a(+) with *ftsZ*_Ana_ inserted in frame between the NdeI-SacI sites. Amp^R^ | This study |
| petFtsZCf-His | pET21a(+) with *ftsZ*_Cf_ inserted in frame between NdeI-XhoI sites. Amp^R^ | This study |
| petFtsZFm-HA | pet21a(+) with *ftsZ*_Fm_-HA inserted between the NheI-EcoRI sites. Amp^R^ | This study |
| pEZGFm | pRL25C with P_petE_::*ftsZ*_Fm_-*gfp* inserted between the BamHI-EcoRI sites. Km^R^, Nm^R^. | This study |
| pEZGAna | pRL25C with P_petE_::*ftsZ*_Ana_-*gfp* inserted between the BamHI-EcoRI sites. Km^R^, Nm^R^. | This study |
| pEZGCf | pRL25C with P_petE_::*ftsZ*_Cf_-*gfp* inserted between the BamHI-EcoRI sites. Km^R^, Nm^R^. | This study |
| pAGZFm | pRL25C with P_glnA_::*gfp*-*ftsZ*_Fm_ inserted between the BamHI-EcoRI sites. Km^R^, Nm^R^. | This study |
| pAGZAna | pRL25C with P_glnA_::*gfp*-*ftsZ*_Ana_ inserted between the BamHI-EcoRI sites. Km^R^, Nm^R^ | This study |
| pAGZCf | pRL25C with P_glnA_::*gfp*-*ftsZ*_Cf_ inserted between the BamHI-EcoRI sites. Km^R^, Nm^R^ | This study |
| pFZGFm | pRL25C with P_ftsZFm_::*ftsZ*_Fm_-*gfp* inserted between the BamHI-EcoRI sites. Km^R^, Nm^R^ | This study |
| pFZGAna | pRL25C with P_ftsZAna_::*ftsZ*_Ana_-*gfp* inserted between the BamHI-EcoRI sites. Km^R^, Nm^R^ | This study |
| pFZGCf | pRL25C with P_ftsZCf_::*ftsZ*_Cf_-*gfp* inserted between the BamHI-EcoRI sites. Km^R^, Nm^R^ | This study |
| pFZHAFm | pRL25C with P_ftsZFm_::*ftsZ*_Fm_-HA inserted between the BamHI-EcoRI sites. Km^R^, Nm^R^ | This study |
| pEGMAna | pRL271 with P_petE_::*gfp*-*mreB*_Ana_ inserted between the XhoI-SacI sites. Cm^R^, Em^R^ | This study |
| pEGMFm | pRL25C with P_petE_::*gfp*-*mreB*_Fm_ inserted between the BamHI-EcoRI sites. Km^R^, Nm^R^ | This study |
| pEGMCf | pRL25C with P_petE_::*gfp*-*mreB*_Cf_ inserted between the BamHI-EcoRI sites. Km^R^, Nm^R^ | This study |
| pEMFm-His | pRL25C with P_petE_::*mreB*_Fm_-His inserted between the BamHI-EcoRI sites. Km^R^, Nm^R^ | This study |
| pPFZGFm | P*_ftsZ_*_Ana_::ftsZ_Fm_-*gfp* inserted as a BglII blunted fragment in SacI blunted pRL271. Cm^R^, Em^R^ | This study |
| pPFZGAna | P_ftsZFm_::*ftsZ*_Ana_-*gfp* inserted as a BglII blunted fragment in SacI blunted pRL271. Cm^R^, Em^R^ | This study |
| pTHS207 | pRL25C; P_mreBAna_::*gfp-mreB*_Ana_ inserted into BamHI and EcoRI-digested pRL25C, Nm^R^, Km^R^ | This study |
| pTHS215 | pRL25C; P_mreBFm_::*gfp-mreB*_Fm_ inserted into BamHI and EcoRI-digested pRL25C, Nm^R^, Km^R^ | This study |

* Modified *gfpmut*3.1 in which the internal NdeI site was removed by replacing CAT by the synonymous CAC codon.

**References**

Black, T. A., Cai, Y. and Wolk, C. P. (1993) ‘Spatial expression and autoregulation of hetR, a gene involved in the control of heterocyst development in Anabaena’, *Molecular Microbiology*. John Wiley & Sons, Ltd (10.1111), 9(1), pp. 77–84. doi: 10.1111/j.1365-2958.1993.tb01670.x.

Stucken, K. *et al.* (2012) ‘Transformation and conjugal transfer of foreign genes into the filamentous multicellular cyanobacteria (subsection V) Fischerella and Chlorogloeopsis’, *Current Microbiology*, 65(5), pp. 552–560. doi: 10.1007/s00284-012-0193-5.

Tolonen, A. C., Liszt, G. B. and Hess, W. R. (2006) ‘Genetic manipulation of Prochlorococcus strain MIT9313: green fluorescent protein expression from an RSF1010 plasmid and Tn5 transposition’, *Applied and environmental microbiology*. 2006/10/13. American Society for Microbiology, 72(12), pp. 7607–7613. doi: 10.1128/AEM.02034-06.

Wolk, C. P. *et al.* (1988) ‘Isolation and complementation of mutants of Anabaena sp. strain PCC 7120 unable to grow aerobically on dinitrogen.’, *Journal of bacteriology*, 170(3), pp. 1239–1244. doi: 10.1128/jb.170.3.1239-1244.1988.
